# Ecological correlates and predictors of Lassa fever incidence in Ondo State, Nigeria 2017–2021: an emerging urban trend

**DOI:** 10.1038/s41598-023-47820-3

**Published:** 2023-11-27

**Authors:** Simeon Cadmus, Olalekan John Taiwo, Victor Akinseye, Eniola Cadmus, Gboyega Famokun, Stephen Fagbemi, Rashid Ansumana, Anddy Omoluabi, Adekunle Ayinmode, Daniel Oluwayelu, Solomon Odemuyiwa, Oyewale Tomori

**Affiliations:** 1 Department of Veterinary Public Health and Preventive Medicine, University of Ibadan, Ibadan, Oyo State Nigeria; 2 Damien Foundation Genomics and Mycobacteria Research and Training Centre, University of Ibadan, Ibadan, Oyo State Nigeria; 3 Centre for Control and Prevention of Zoonoses, Faculty of Veterinary Medicine, University of Ibadan, Ibadan, Oyo State Nigeria; 4 Department of Geography, University of Ibadan, Ibadan, Oyo State Nigeria; 5 Department of Chemical Sciences, Augustine University, Ilara-Epe, Lagos State Nigeria; 6 Department of Community Medicine, College of Medicine, University of Ibadan, Ibadan, Oyo State Nigeria; 7 Department of Epidemiology and Disease Control, Ondo State Ministry of Health, Ondo State, Nigeria; 8 School of Community Health Sciences, Njala University, Bo, Sierra Leone; 9 Peltom Global Services, Abuja, Nigeria; 10 Department of Veterinary Parasitology and Entomology, University of Ibadan, Ibadan, Oyo State Nigeria; 11 Department of Veterinary Microbiology, University of Ibadan, Ibadan, Oyo State Nigeria; 12 Department of Veterinary Pathobiology, University of Missouri, Columbia, MO USA; 13 African Centre of Excellence for Genomics of Infectious Diseases, Redeemer’s University, Ede, Osun State Nigeria

**Keywords:** Ecology, Microbiology, Ecology, Diseases, Medical research, Risk factors

## Abstract

Lassa fever (LF) is prevalent in many West African countries, including Nigeria. Efforts to combat LF have primarily focused on rural areas where interactions between rodents and humans are common. However, recent studies indicate a shift in its occurrence from rural to urban areas. We analysed secondary data of reported LF outbreaks from 2017 to 2021 in Ondo State, Nigeria to identify the distribution pattern, ecological variations, and other determinants of disease spread from the ward level using nearest neighbour statistics and regression analysis. Data utilised include LF incidence, ecological variables involving population, nighttime light intensity, vegetation, temperature, market presence, road length, and building area coverage. ArcGIS Pro 3.0 software was employed for spatial analysis. Results revealed spatio-temporal clustering of LF incidents between 2017 and 2021, with an increasing trend followed by a decline in 2021. All wards in Owo Local Government Area were identified as LF hotspots. The ecological variables exhibited significant correlations with the number of LF cases in the wards, except for maximum temperature. Notably, these variables varied significantly between wards with confirmed LF and those without. Therefore, it is important to prioritise strategies for mitigating LF outbreaks in urban areas of Nigeria and other LF-endemic countries.

## Introduction

Lassa fever (LF) is an acute and sometimes severe viral haemorrhagic illness transmitted by rodents^1^. It is endemic in West Africa, particularly rural Nigeria and the Mano River Union countries (Liberia, Guinea, and Sierra Leone)^1,2^. Discovered in 1969, LF is a threat to human health, accounting for between 300,000 and 500,000 cases and 5000 deaths annually in West Africa, with case fatality rate (CFR) of 15–25% among hospitalised patients^2,3^. The disease presents with various clinical manifestations, including several cases thought to be mild or asymptomatic^4^. Unspecific fever and malaise are the symptoms associated with most cases. However, occasional progression to haemorrhagic symptoms and fatalities in about 20% of severe cases are often observed^4^.

Lassa fever is caused by a single-stranded RNA virus belonging to the Arenaviridae family^5^. There are currently six main Lassa virus (LASV) clades distributed across different West African countries: clades I–III (Nigeria), clade IV (Sierra Leone, Guinea, and Liberia), clade V (southern Mali) and clade VI (recently reported from Togo). The sustenance of LASV transmission has been attributed to its ability to mutate over time^6^. Lassa fever poses significant public health challenge with recurrent yearly seasonal outbreaks in West Africa ^4^. Although the disease is geographically associated with some specific countries in West Africa, it is one of the most exported viral haemorrhagic fevers (VHFs)^7^. In this sub-region alone, it is estimated that LF affects about 2 million persons, resulting in about 5000–10,000 deaths annually^8^. Two major epicentres of the disease have been identified in West Africa, the first in Nigeria and the second localised around Sierra Leone and Liberia^8^. However, recent sporadic outbreaks have been widespread around most countries in the sub-region, particularly in Nigeria^5^.

Several factors have been identified as responsible for exacerbating and sustaining the transmission of LF in Nigeria. Some of these include food exposure to droppings or urine of infected rodents that normally occur during open drying of grains and other food items^9^. Other factors are the consumption of infected rodents, inappropriate practices such as unhygienic waste disposal and poor environmental sanitation^9–11^. It has been reported that the burden of LF is more associated with the rural areas owing to the preponderance of associated risk factors in these settings, some of which include cultural practices (hunting, processing and consumption of rodents as protein source, and drying of food items by the roadside) and poverty^12–15^. However, recent studies have reported a probable shift in LF occurrence from rural to main urban or urban slum settings^12,14^.

Currently, in Nigeria, 5083 suspected cases of LF were reported between January and May 2023, with 1067 (13.0%) confirmed and 156 deaths (CFR = 17.7%)^16^. Overall, 112 Local Government Areas (LGAs) across 27 States of the country reported at least one confirmed case over this period in 2022. Furthermore, three states accounted for about 72% of the cases reported in 2022 (Ondo: 33%, Edo: 25% and Bauchi: 14%)^17^. Thus, for the first time since 2017, Ondo State surpassed Edo State as the epicentre of LF in Nigeria^18^. The steady increase in the number of cases of LF in Ondo State underscores the importance of the state in the overall epidemiology of LF in Nigeria, and this poses huge concerns for public health.

Additionally, this increase highlights the need for further investigation of the dynamics of occurrence and spread of LF in community settings in Ondo State, Nigeria. Although previous workers studied the effects of seasonal variations^19^ as well as climatic parameters such as rainfall and temperature^8,20^ on the incidence of LF, little has been done to assess the impact of multiple ecological indicators on the incidence of the disease at the smallest administrative units (i.e., ward level) in a hotspot location such as Ondo State. This study was therefore designed to determine the ecological correlates and predictors of LF in Ondo State over five years (2017 to 2021) with a view to examining the distribution pattern of persons diagnosed with LF, as well as the correlates and determinants of LF incidence and occurrence at the ward level in the state.

## Results

### Spatio-temporal distribution of Lassa fever incidents between 2017 and 2021

**Figure 1:**
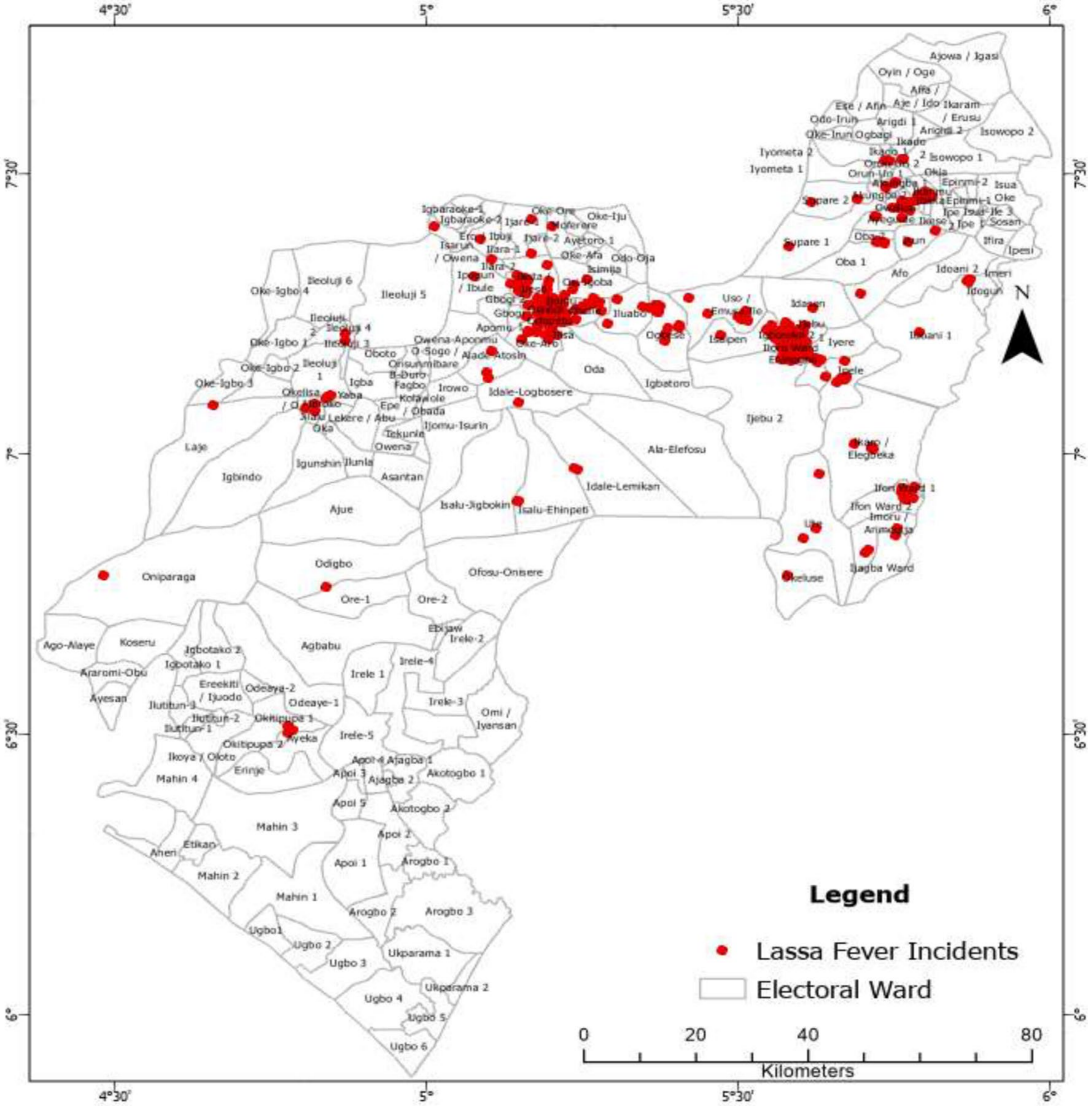
Spatial distribution of Lassa fever incidents between 2017 and 2021 in Ondo State.

A list of 4315 suspected LF cases from 2017 to 2021 was obtained, including 1057 laboratory-confirmed cases (24.50% of all cases) (Fig. 1). The remaining cases were negative or classified as probable. Furthermore, out of the individuals included in the data, 821 individuals (77.62%) were still alive at the time of data collection.

The number of confirmed LF incidents increased significantly from 61 in 2017 to 147 in 2018 (140.98% increase). Subsequently, there were increases of 82.31% in 2019 and 54.48% in 2020. However, there was a notable decline of 59.66% between 2020 and 2021 (Supplementary Fig. 1). Regarding temporal incidence, the highest number of confirmed cases occurred in 2020 (39.17%), followed by 2019, 2021, 2018, and 2017. These findings align with a study by Dalhat et al. (2022)^9^ that analysed LF surveillance data from all states in Nigeria between January 2018 and December 2021.

Notably, the temporal analysis of LF incidents in Ondo State from 2017 to 2021 revealed significant spatial clustering. The Average Nearest Neighbour Statistic values (ranging from 0.161168 to 0.376471) indicated clustering in specific locations rather than random dispersion across the state (Table 1). Kernel Density Estimation (KDE) results showed that the majority of LF clusters were concentrated in Owo town, while Akure town had a notable number of cases but no detected clusters. This suggests a random distribution in Akure town.

**Table 1:** Temporal patterns of LF incidents among wards in Ondo State.

Year	Average nearest neighbour statistic				
	No of incidents	Observed mean distance	Expected mean distance	Z-score	Nearest neighbour statistics (Rn)
2017	61	2152.47	5717.49	9.32	0.376471
2018	147	577.42	3582.71	19.46	0.161168
2019	268	833.39	3280.85	23.36	0.254017
2020	414	756.79	2793.51	28.38	0.270911
2021	167	1262.64	4267.67	17.41	0.295862
2017–2021	1057	324.64	1826.44	51.14	0.177745

### Spatiotemporal pattern of Lassa fever incidents among wards between 2017 and 2021

Out of the 203 wards in Ondo State, 70 wards (34.48%) experienced LF incidents between 2017 and 2021 (Supplementary Table 1). The distribution of incidents varied across the years, with an increase in the number of affected wards. In 2017, there were 26 wards with LF incidents, which increased to 28 in 2018, 45 in 2019, 55 in 2020, and 35 in 2021. Among the wards with the highest number of incidents, Ehinogbe ward had the most cases in 2017, while Igboroko 2 had the highest in 2018. In 2019, both Ijebu 1 and Ijebu 2 wards had the highest number of incidents. Igboroko 2 ward consistently had the highest number of new cases in subsequent years, followed by Ijebu 2 and Ijebu 1 (Supplementary Table 1). These three wards, Igboroko 2, Ijebu 2, and Ijebu 1, were identified as the major hotspots of LF in Ondo State from 2018 to 2021.

**Figure 2:**
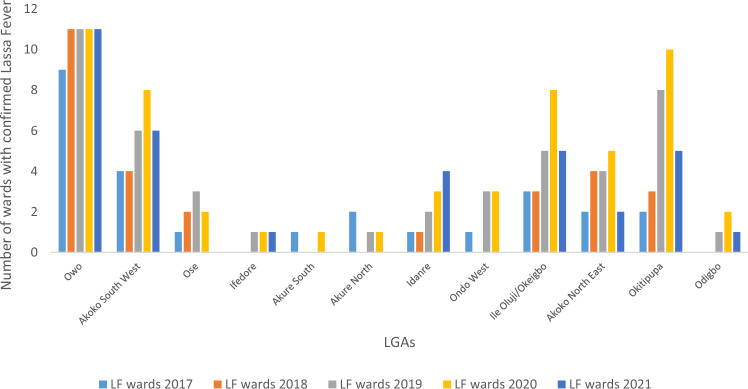
Temporal increase in the number of wards with confirmed LF between 2017 and 2021.

LF was reported in 12 (66.7%) out of 18 LGAs in Ondo State. Owo LGA had the highest number of confirmed LF (651) incidents and was followed by Akoko Southwest (83) and Ose (77) LGAs (Table 2). Owo and Akoko Southwest LGAs had the largest numbers of wards with confirmed LF, while Ose and Akure South LGAs had nine wards with confirmed LF (Table 2). Figure 2 shows the temporal increase in the number of wards with confirmed LF between 2017 and 2021.

**Table 2:** Number of laboratory confirmed LF in the affected LGAs in Ondo State between 2017 and 2021.

LGA	LF2017	LF2018	LF2019	LF2020	LF2021	Total	No of affected wards
Owo	38	104	149	250	110	651	11
Akoko Southwest	2	6	25	40	10	83	12
Ose	6	13	23	23	12	77	9
Ifedore	3	7	21	32	11	74	7
Akure South	3	6	13	38	11	71	9
Akure North	4	9	20	19	9	61	5
Idanre	1	0	6	4	0	11	4
Ondo West	1	2	4	3	0	10	5
Ile Oluji/Okeigbo	2	0	4	1	0	7	3
Akoko Northeast	0	0	2	2	3	7	2
Okitipupa	0	0	1	1	1	3	1
Odigbo	1	0	0	1	0	2	2
Total	61	147	268	414	167	1057	70

In 2017, 2019, 2020, and 2021, wards with similar LF incidents showed clustering, supported by statistical measures. For 2017, the z-score was 4.798255 with a Moran's Index of 0.862321; for 2019, a z-score of 2.238864 and Moran's Index of 0.24755; for 2020, a z-score of 2.396517 and Moran's Index of 0.20656; for 2021, a z-score of 2.810033 and Moran's Index of 0.322375; and for the years 2017 to 2021, a z-score of 5.131246 and Moran's Index of 0.405904 (Table 3). These measures indicate that the observed clustering patterns were not likely due to chance. However, in 2018, the LF incidents among wards followed a random pattern without statistical significance. The high z-score and low pseudo p-value provided strong evidence of the statistical significance of the calculated Global Moran's I, confirming that wards with similar LF incidents exhibited significant spatial contiguity in Ondo State.

**Table 3:** Global Moran’s index of LF among wards in Ondo State between 2017 and 2021.

Years	Moran's index	Expected index	Variance	Z-score	P-value	Interpretation
2017	0.862321	0.04	0.035364	4.798255	0.000002	Clustered
2018	0.184415	0.037037	0.022561	1.47435	0.140387	Random
2019	0.24755	0.022727	0.014574	2.238864	0.025165	Clustered
2020	0.20656	0.018519	0.008821	2.396517	0.016552	Clustered
2021	0.322375	0.029412	0.015672	2.810033	0.004954	Clustered
2017–2021	0.405904	0.014493	0.006712	5.131246	0.000000	Clustered

### Hotspot of Lassa fever among wards in Ondo State

According to Fichet-Calvet et al. (2014) ^8^ and Redding et al. (2021)^19^, LF prevalence in rodents can vary significantly at small geographic scales, such as neighbouring villages, suggesting a localised and discontinuous LF risk. To identify these localised patterns, Local Indicators of spatial autocorrelation were used. In Ondo State, the northeastern section, particularly in Owo town, was identified as the main hotspot for LF incidents. In 2017, eight wards were identified as hotspots, which reduced to six wards in 2018, with new additions and exclusions. Overall, 11 wards consistently appeared as hotspots between 2017 and 2021 (Table 4). The hotspot maps from 2017 to 2021 display the concentration of LF incidents in these areas (Supplementary Figs. 2A–F). The hotspots were predominantly located in Owo LGA, except for Ogbese ward in Akure North LGA. This pattern persisted, with all wards in Owo LGA (except Uso/Emure-Ile) and Ogbese ward in Akure South LGA identified as significant LF hotspots when considering the entire dataset from 2017 to 2021.

**Table 4:** Wards with significant hotspots of LF between 2017 and 2021 in Ondo State.

Hotspot 2017	Hotspot 2018	Hotspot 2019	Hotspot 2020	Hotspot 2021	Hotspot 2017–2021
Ehinogbe	Idasen	Ehinogbe	Ehinogbe	Ehinogbe	Ehinogbe
Ogbese	Igboroko 1	Idasen	Idasen	Idasen	Idasen
Igboroko 1	Igboroko 2	Igboroko 1	Igboroko 1	Igboroko 1	Igboroko 1
Igboroko 2	Ijebu 1	Igboroko 2	Igboroko 2	Igboroko 2	Igboroko 2
Ijebu 1	Isaipen	Ijebu 1	Ijebu 1	Ijebu 1	Ijebu 1
Ijebu 2	Iyere	Ijebu 2	Ijebu 2	Ijebu 2	Ijebu 2
Iloro		Iloro	Iloro	Iloro	Iloro
Isaipen		Ipele	Isaipen	Isaipen	Ipele
		Isaipen	Iyere	Iyere	Isaipen
		Iyere	Ogbese	Ogbese	Iyere
					Ogbese

### Correlates and variations in the LF incidents across ecological and demographical factors in Ondo State

The study utilised the urban and rural classification of wards by Grid3.org to analyse the difference in the number of confirmed LF cases in Ondo State. Between 2017 and 2021, the average number of LF incidents in rural wards (3.85 ± 14.068) was significantly lower than in urban wards (16.95 ± 36.411) (t(201) = 4.289, p = 0.000). The area occupied by buildings in wards with LF incidents (856,774.6609 ± 1,352,574.833) was significantly higher than in wards without LF incidents (284,858.9932 ± 390,088.3419) (t(201) = 4.550, p = 0.000), showing a positive correlation between LF cases and building area (r = 0.467, p = 0.000). Similarly, the number of buildings in wards with LF incidents (5469.94 ± 8119.98) was significantly higher than in wards without LF incidents (2164.84 ± 2676.94) (t(201 = 4.289, p = 0.000), correlating positively with LF cases (r = 0.409, p = 0.000). Additionally, the population in wards with LF incidents (26,771.84 ± 37,788.64) was significantly higher than in wards without LF incidents (11,294.58 ± 14,107.78) (t(201) = 4.213, p = 0.000), with a positive correlation between LF incidents and ward population (r = 0.400, p = 0.000). These findings indicate that higher building area, number of buildings, and population are associated with a higher number of confirmed LF cases.

The average elevation in wards with confirmed LF incidents (299.77 ± 99.69 m) was significantly higher than in wards without LF incidents (213.65 ± 166.87 m) (t(201) = 4.289, p = 0.000), and there was a positive correlation between LF cases and elevation (r = 0.232, p = 0.000). Furthermore, the average Normalised Difference Vegetation Index (NDVI) in wards with confirmed LF incidents (0.328425 ± 0.105014) was significantly lower compared to wards without LF incidents (0.378117 ± 0.056094) (t(201) = 4.399, p = 0.000), with a negative correlation between LF cases and NDVI (r = − 0.254, p = 0.000). Additionally, the average Nighttime Light (NTL) in wards with confirmed LF incidents (0.715723 ± 0.778167) was significantly higher than in wards without LF incidents (0.321713 ± 0.138504) (t(201) = 5.701, p = 0.000), and there was a positive correlation between LF cases and NTL (r = 0.343, p = 0.000). These findings indicate that higher elevation and NTL, as well as lower NDVI, are associated with a higher number of confirmed LF cases.

The average length of roads in wards with confirmed LF incidents (92.96 ± 109.53 km) was significantly higher than in wards without LF incidents (43.19 ± 45.73 km) (t(201) = 4.553, p = 0.000), and there was a positive correlation between LF cases and road length (r = 0.300, p = 0.000). Moreover, the average number of markets in wards with confirmed LF incidents (1.03 ± 1.32) was significantly higher than in wards without LF incidents (0.57 ± 0.78) (t(201) = 3.134, p = 0.001), and there was a positive correlation between LF cases and the number of markets (r = 0.191, p = 0.003). This suggests that as the number of markets increases, there is a slight increase in LF cases. Similarly, the average distance to markets in wards with confirmed LF incidents (4279.62 ± 3176.54 m) was significantly lower than in wards without LF incidents (6537.40 ± 5867.84 m) (t(201) = 2.977, p = 0.001), and there was a negative correlation between LF cases and distance to markets (r = − 0.223, p = 0.001).

The average minimum temperature in wards with confirmed LF incidents (22.05 ± 0.59 °C) was significantly lower compared to wards without LF incidents (22.47 ± 1.08 °C) (t(201) = 3.053, p = 0.000). There was a negative and significant correlation between LF cases and minimum temperature (r = − 0.189, p = 0.003). However, the average maximum temperature did not significantly differ between wards with confirmed LF incidents (31.58 ± 0.33 °C) and wards without LF incidents (31.51 ± 0.35 °C) (t(201) = 1.367, p = 0.506), and there was no significant relationship between LF cases and maximum temperature (r = − 0.064, p = 0.183). On the other hand, the average precipitation in wards with confirmed LF incidents (1546.83 ± 174.27 mm) was significantly lower compared to wards without LF incidents (1833.99 ± 388.64 mm) (t(201) = 5.838, p = 0.000), and there was a negative and significant correlation between LF cases and precipitation (r = − 0.324, p = 0.000).

**Figure 3:**
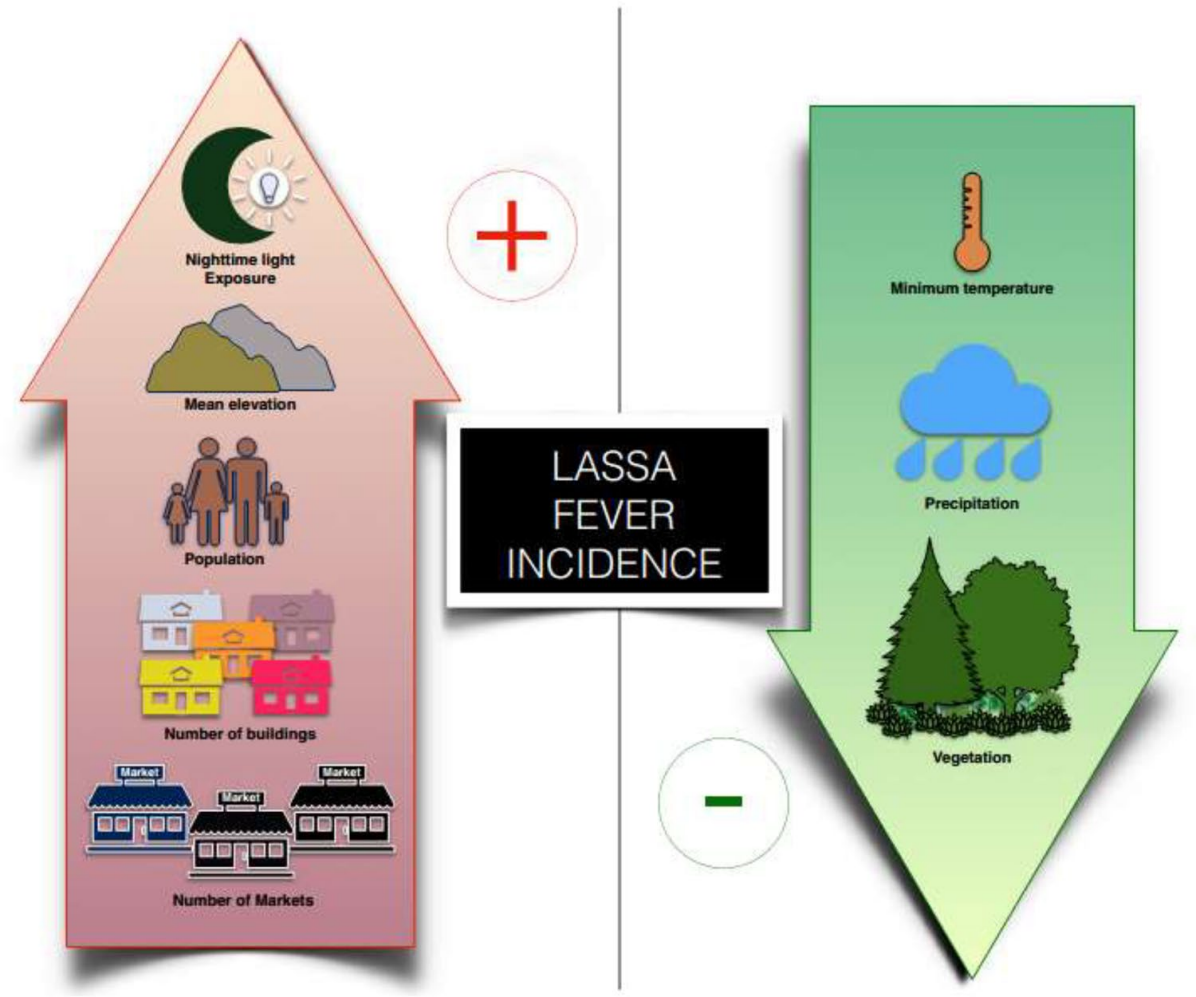
Relationship between some ecological and demographical factors and confirmed LF incidents between 2017 and 2021 in Ondo State.

Figure 3 presents a summary of the relationship between LF cases and number of markets, population, mean elevation, number of buildings, nighttime exposure, minimum temperature, precipitation and vegetation.

### Spatial predictors of Lassa fever incidents among wards in Ondo state

Principal Component Analysis (PCA) was used to address high multicollinearity (VIF > 10) among predictor variables. Three uncorrelated factors were extracted, explaining 86.18% of the total variance. The first factor, labeled "Human factor," included variables like buildings, area occupied by buildings, population, and length of roads. The second factor, "Physical factor," comprised average elevation, temperature, and precipitation. The third factor, "Environmental factor," included NDVI and NTL. These factors collectively captured 86.18% of the original predictors' information, with the first factor explaining 34.53%, the second factor 32.78%, and the third factor 17.08% of the variance.

The relationship between the number of confirmed LF cases and the extracted factor scores was examined using Ordinary Least Square (OLS) regression. The three factors accounted for only 15.4% of the variation in LF incidents, assuming constant relationships across the study area. To relax this assumption, Geographically Weighted Regression (GWR) with an optimal bandwidth of 50 nearest neighbors was employed. Multiscale Geographical Weighted Regression (MGWR) was then used to identify LF predictors in each ward.

Supplementary Table 2 presents the results, showing that the R-Square increased from 15.4% in OLS to 67.34% in GWR, and further to 71.90% in MGWR. The Adjusted R-Square coefficient was highest for MGWR, indicating a better model fit. Additionally, MGWR had the lowest Akaikes Information Criterion (AICc) value, suggesting its effectiveness in modeling the spatially varying relationship.

The coefficient estimates from Table 5 reveal that human factors and environmental factors are positively correlated with LF incidences, while physical factors show a negative correlation. Human factors exhibit the strongest positive association with LF incidence and display more spatial variations compared to environmental or physical factors.

**Table 5:** Summary statistics for coefficients estimates.

Explanatory variables	Mean	Standard deviation	Minimum	Median	Maximum
Intercept	0.501	0.8615	− 0.4095	0.2471	1.5297
Human factor	1.389	1.1657	0.1446	0.3768	2.8589
Physical factor	− 0.1854	0.0273	− 0.2325	− 0.1956	− 0.1247
Environmental factor	0.5916	0.4881	0.0966	0.2106	1.2121

Supplementary Table 3 which is model diagnostic coefficients showed that MGWR had slightly lower R-Squared coefficients compared to GWR, but MGWR had a lower AICc (165.17) and higher Adjusted R-Squared (0.5054) than GWR (171.00 and 0.4875), respectively. Therefore, MGWR was selected to model the association between human, physical, and environmental factors and LF incidence across wards in Ondo State.

Table 6 which is summary of explanatory variables and neighborhoods reveals that none of the independent variables operated locally. Human and environmental factors operated regionally, while physical factors operated globally. Human factors had a broader association with LF incidence across wards compared to environmental factors.

**Table 6:** Summary of explanatory variables and neighborhoods.

Explanatory variables	Neighbors (% of features)^a^	Significant (% of features)^b^
Intercept	39 (55.71)	54 (77.14)
Human factor	36 (51.43)	60 (85.71)
Physical factors	70 (100.00)	0 (0.00)
Environmental factors	45 (64.29)	34 (48.57)

^a^This number in the parenthesis ranges from 0 to 100%, and can be interpreted as a local, regional, global scale based on the geographical context from low to high.

^b^In the parentheses, the percentage of features that have significant coefficients of an explanatory variable.

In Supplementary Fig. 3A, wards shaded deep brown displayed a strong, significant positive relationship with the number of LF incidents, while some orange-colored wards also had a positive relationship. None of the wards showed a negative relationship with human-related factors. A total of 34 wards exhibited a positive association, with 25 wards having a strong positive relationship. In Supplementary Fig. 3B, no significant association was found between LF incidents and physical factors. Supplementary Fig. 3C showed that environmental factors had a strong, positive association with LF incidents in 23 wards and a significant association in six wards, while Supplementary Fig. 3D displayed a similar pattern for the intercept.

There were 35 wards where a strong, significant positive relationship existed between the number of confirmed LF incidents and human and environmental factors (Supplementary Fig. 3E). This indicates that as the human and environmental factors increased, the number of confirmed LF incidents also increased among the wards in Ondo State.

### Predictors of the likelihood of confirmed LF cases among wards in Ondo State

The analysis in Table 7 revealed significant associations between various factors and the number of LF incidents in wards. Nighttime light intensity showed a positive relationship, with each unit increase corresponding to an increase of 3.150 wards with confirmed LF incidents. Wards with high nighttime light intensity had 23.335 times higher odds of having confirmed LF incidents compared to those with low intensity. Longer road lengths were also positively associated with LF incidents, with each unit increase resulting in an increase of 0.012 wards. Wards with longer road lengths had 1.012 times higher odds of having confirmed LF incidents. Higher maximum temperatures were positively correlated with LF incidents, with each unit increase associated with an increase of 2.773 wards. Wards with higher temperatures had 16.006 times higher odds of having confirmed LF incidents. Conversely, higher precipitation was negatively associated with LF incidents, with each unit increase leading to a decrease of 0.006 wards. Wards with higher precipitation had 0.994 lower odds of having confirmed LF incidents.

**Table 7:** Predictors of LF at ward level.

	Variables	B	S.E	Wald	Df	Sig	Exp(B)	95% C.I. for EXP(B)	
								Lower	Upper
Step 1^a^	Precipitation	− 0.003	0.001	24.723	1	0.000	0.997	0.996	0.998
	Constant	4.356	0.984	19.596	1	0.000	77.924		
Step 2^b^	Maximum Temperature	4.002	0.765	27.349	1	0.000	54.729	12.212	245.281
	Precipitation	− 0.006	0.001	39.291	1	0.000	0.994	0.992	0.996
	Constant	− 116.221	23.039	25.448	1	0.000	0.000		
Step 3^c^	Length of Roads	0.011	0.004	8.845	1	0.003	1.011	1.004	1.019
	Maximum Temperature	3.538	0.764	21.418	1	0.000	34.401	7.688	153.924
	Precipitation	− 0.007	0.001	37.042	1	0.000	0.993	0.991	0.996
	Constant	− 101.997	23.017	19.637	1	0.000	0.000		
Step 4^d^	NightTime Light	3.150	1.294	5.926	1	0.015	23.335	1.848	294.701
	Length of Roads	0.012	0.004	9.548	1	0.002	1.012	1.004	1.020
	Maximum Temperature	2.773	0.793	12.240	1	0.000	16.006	3.386	75.674
	Precipitation	− 0.006	0.001	25.083	1	0.000	0.994	0.992	0.997
	Constant	− 80.899	23.808	11.546	1	0.001	0.000		

## Discussion

This study addresses the gap in research on LF by focusing on micro-scale analysis at the ward level in Nigeria. Previous studies have predominantly examined LF at regional or national scales, potentially overlooking the intricate interplay of human, physical, and environmental factors that sustain LF incidents. By conducting a comprehensive spatial analysis, this study determined the spatial patterns of LF incidents and identified significant differences in ecological factors between wards with and without LF cases. Furthermore, the study identified the correlates and predictors of LF across wards in Ondo State, Nigeria. Unlike previous research, which primarily considered rainfall, temperature, vegetation, and altitude, this study incorporates additional factors such as population distribution, market numbers, distance from markets, and NTL intensity to assess the likelihood of LF incidents in the study area, expanding the understanding of LF dynamics.

During the study period, there was a notable increase in confirmed LF cases in Ondo State, Nigeria perhaps due to improved surveillance and disease reporting. However, the possible role of poor sanitation, insufficient healthcare infrastructure, limited access to diagnostics, inadequate infection prevention and control practices, and low level of awareness among the general population cannot be discountenanced^21,22^. With its substantial rural population engaged in activities like farming and hunting, Ondo State faces an elevated risk of LF due to continuous exposure to rodents. Moreover, healthcare facilities in certain areas of Ondo State, like Owo and Ose LGAs, lack essential resources for infection prevention and control^23^. Media campaigns targeting LF awareness have had limited impact in rural regions, resulting in insufficient knowledge of preventive measures and curative practices^24^. Similar challenges have been observed in Akwa Ibom, indicating the need for improved health behaviour modifications following health campaigns^25^.

The occurrence of LF showed significant temporal and spatial clustering in Ondo State, Nigeria. The highest LF incidence was observed in Owo LGA, followed by Akoko Southwest LGA, located northward and adjacent to Owo LGA. Additionally, Ose LGA, situated eastward of Owo LGA, reported many LF cases. These findings support previous studies and suggest the possibility of human-to-human transmission in the contiguous LGAs^25,26^. LF diagnoses were concentrated in specific localities and wards, particularly in indigenous areas of towns, indicating factors such as poor hygiene, inadequate waste management, limited healthcare access, population density, and poor ventilation as potential contributors to LF epidemics in Ondo State. The maximum clustering of LF cases occurred within a distance of 20,800 m, highlighting the area with the most pronounced spatial processes promoting clustering (Supplementary Plates 1 and 1).

The clustering of LF in traditional areas of cities can be attributed to various factors, including environmental, social, and cultural influences. Environmental factors such as land use and vegetation cover affect the ecology of rodents, which serve as the primary reservoirs for the Lassa virus. Traditional areas often have open spaces used as waste dumpsites, buildings that provide access points for rodents, and less developed infrastructure. Poor household waste management practices and close-knit communities in these areas can contribute to LF clustering. Limited access to healthcare services in traditional areas may lead residents to rely on traditional healers or self-medication. Addressing LF clustering in traditional areas requires a comprehensive understanding of the complex interactions between environmental, social, and cultural factors. Urban ecological factors such as population density, land use patterns, and sanitation conditions play a role in LF incidents. High population density and poor sanitation can create favorable conditions for rodent populations, increasing the risk of LF outbreaks (12, 13, 14, 15). Housing characteristics and domestic practices also influence rodent density, potentially affecting LF transmission to humans^27^.

Between 2017 and 2021, 10 wards in Owo LGA (Ehinogbe, Idasen, Igboroko 1, Igboroko 2, Ijebu 1, Ijebu 2, Iloro, Ipele, Isaipen, and Iyere) and one ward in Akure North LGA (Ogbese) were identified as LF hotspots. LF incidents in other wards in Akure appeared as outliers without significant hotspots. The observed hotspots in specific wards may be attributed to several factors. Favorable environmental conditions in wards with suitable rodent habitats can facilitate virus transmission. Poor sanitation and waste management practices can serve as breeding grounds for rodents, increasing the risk of human exposure^9–11^. Social and cultural factors, such as traditional food storage practices and communal living arrangements, may also contribute to the clustering of cases in certain areas^12–15^. Limited access to healthcare services and delays in diagnosis and treatment can worsen the impact of the disease. Variations in surveillance and reporting practices across communities can affect LF case detection and reporting, leading to apparent hotspots in wards with more rigorous surveillance and reporting systems, particularly in areas with a history of LF prevalence^23^.

Lassa fever is an endemic viral illness in Nigeria, with a growing prevalence in urban areas due to the expansion of cities, changes in land use, and shifts in population demographics^28,29^. Higher population densities in urban areas may facilitate human-to-human transmission and impact the distribution of rodent reservoirs. Additionally, environmental conditions in urban areas differ from rural areas, influencing the abundance of virus-carrying rodents^29^. This study reveals that urban wards in Ondo State had a higher number of LF cases compared to rural wards, in contrast to a study by Gomerep et al. (2022) conducted in Plateau State. These findings suggest a complex relationship between residence and LF incidence, emphasising the need for further investigation^12^.

Urban indicators, including the number of buildings, population size, presence of markets, proximity to markets, NTL, road length, and lower vegetation (NDVI), are closely linked to higher incidents of LF across wards^8,30–33^. These highlight LF as a growing urban health concern in Ondo State^12^. Redding et al. (2021) similarly found a positive association between LF and urbanisation, specifically measured by built-up areas, which may impact human-rodent interactions^19^.

Lassa fever incidents were found to be more common in wards with higher elevation, minimal rainfall, and lower minimum temperature^19,20^. The significant predictors of LF incidents in Ondo State's wards were human factors such as the number of buildings, population size, and road length, along with environmental factors like NDVI and NTL intensity. However, physical factors like average elevation, temperature, and precipitation did not play a significant role in explaining the number of LF incidents. These align with Fichet-Calvet and Rogers' (2009) previous findings, which suggested that neither vegetation nor elevation had a strong predictive value for LF^8^.

The occurrence of LF incidents in Ondo State's wards is influenced by factors such as NTL intensity, road length, maximum temperature, and precipitation. These findings are consistent with previous studies^8^ that have shown a negative association between LF cases and minimum temperature. There is evidence suggesting that NTL exposure may affect rodent abundance. Artificial lighting can disrupt rodent behavior, attract them to lit areas for foraging and nesting, and potentially make them more susceptible to Lassa virus infection. Additionally, light pollution's impact on melatonin production, a hormone regulating circadian rhythms and immune response, may further contribute to rodent vulnerability^34,35^.

The increasing prevalence of exposure to light at night has significant social, ecological, behavioral, and health consequences that are only now becoming apparent^36^. The presence of artificial light at night disrupts the natural behavior of rodents, attracting them to illuminated areas for foraging and nesting^34^. Higher levels of NTL exposure in urban areas are associated with increased LF incidence, potentially due to increased rodent abundance and altered behavior patterns^34,35^. This suggests that NTL exposure may contribute to the urbanisation of LF. However, further research is needed to fully understand the relationship between NTL exposure, rodent abundance, and LF transmission. Road length is believed to be associated with LF incidence, as it influences factors such as changes in land use patterns, human activity, and rodent reservoir distribution^27^. Roads also facilitate the movement of humans, potentially aiding the spread of the virus across regions and populations.

Lassa fever is a virus that is sensitive to changes in temperature and humidity, and outbreaks have been linked to these conditions. For instance, droughts or extreme weather events can cause rodents to move into urban areas, thereby increasing the risk of human exposure^37^. The relationship between temperature and LF incidence is complex and may be influenced by other factors, such as rainfall, humidity, and land use patterns. In Sierra Leone, LF incidence shows seasonal fluctuations, with peaks during the dry season and smaller peaks during the rainy season, indicating that climatological factors and agricultural labor patterns may impact rodent-human contact^38^. Fichet-Calvet and Rogers (2009) demonstrated that recorded LF outbreaks in human populations occurred in areas receiving annual rainfall between 1500 and 3000 mm^8^. Changes in temperature can affect the immune response of humans and rodents, potentially influencing the severity and outcomes of LF infections^39^. Temperature plays a crucial role in the transmission and incidence of LF, which is primarily carried by rodents and transmitted to humans through contact with contaminated excreta or urine. There is evidence suggesting that higher temperatures may be associated with increased LF incidence, with the highest incidence observed during the hottest months of the year^40^. Higher temperatures can enhance the reproductive rates of rodent populations, leading to an increase in the number of infected animals and potentially raising the risk of transmission to humans^39^. Understanding these temperature-related dynamics can help shape strategies aimed at reducing the urbanisation of LF and mitigating the risk of outbreaks in urban areas.

The major limitation of this study is the use of secondary data which may have limited the scope of interpretation of results. Nevertheless, the remotely sensed secondary data used were the most adequate in a data-scarce environment like Nigeria. Moreover, our findings provided valuable insight into the spatial factors that contributed to the increased incidence of urban LF cases in the study area.

## Conclusion

Lassa fever incidence in Ondo State, Nigeria has been on the increase since 2017 with significant clusters noticed within urban rather than rural wards. The clusters were most prominent in the city core where hygiene and access to healthcare facilities are poor, thereby providing a favourable habitat where *Mastomys* rodents may thrive. Ecological factors such as population density, land use patterns, and sanitation conditions favour increased LF incidence in urban compared to rural wards. Human and environmental factors such as nighttime light intensity and length of roads positively influenced the number of confirmed LF cases in wards in Ondo State. Hence, strategies to prevent and control LF outbreaks in urban areas such as improved sanitation, proper waste management, implementation of effective rodent control measures and public health education are advocated.

## Methods

### Study area

Ondo State, situated in southwestern Nigeria, shares borders with Ekiti, Kogi, Osun, Ogun, and Edo States. It has an estimated population of approximately 4.6 million people. The people in Ondo State are engaged in diverse occupations, with significant number involved in agriculture, particularly farming and fishing, due to the State's coastal location. The economy of Ondo State is primarily agrarian, with agriculture playing a pivotal role. Moreover, the State is renowned for producing crops such as cocoa, palm oil, yam, maize, and cassava. Ondo State experiences a tropical climate with two distinct seasons. The rainy season spans from April to October, characterised by heavy rainfall, while the dry season lasts from November to March, featuring reduced rainfall and higher temperatures. The State has a well-developed road network that connects its cities, towns, and rural areas. Major roads include the Akure-Owo Road, Akure-Ondo Road, and the Akure-Ilesha Expressway. These roads facilitate transportation within the state and connect it to other states in Nigeria.

### Data sources

Hospital records from two specialised healthcare facilities, covering 2017 to 2021, were utilised. However, these records did not include coordinates of patients' house addresses; instead, descriptive addresses were provided. The study assumed the patient's home as the point of contracting and spreading the infection. Google Earth was used to georeference the addresses in the patient’s files because of its comprehensive road and street address data on Ondo State, allowing a near precise location of individual addresses using street names. This approach facilitated accurate mapping and ensured confidentiality of patient identity. The coordinates of all individuals were extracted from Google Earth and mapped using ArcGIS Pro 3.0 software. Since the raw data included the number of individuals with LF symptoms, laboratory confirmed cases were filtered for analysis. All spatial data used in the analysis were projected to the Universal Transverse Mercator (UTM) Zone 31 for precise computation of parameters.

Previous research on LF has primarily focused on national or Local Government Area units, with limited attention to ward-level patterns^19,41^. In Nigeria, the ward represents the smallest administrative unit. However, the lack of accessible and available ward boundary data has hindered spatial analysis. To address this, the digital administrative ward boundary for Ondo State was downloaded from https://grid3.org/ in ArcGIS Pro compatible shape-file format. The downloaded ward boundary file included additional attributes such as ward area, classification (urban/rural), and unique identifiers. The ward boundary served as the basis for aggregating and summarising all other data related to LF analysis in Ondo State. Consequently, the ward boundary was adopted as the analysis and reporting unit for this study.

**Figure 4:**
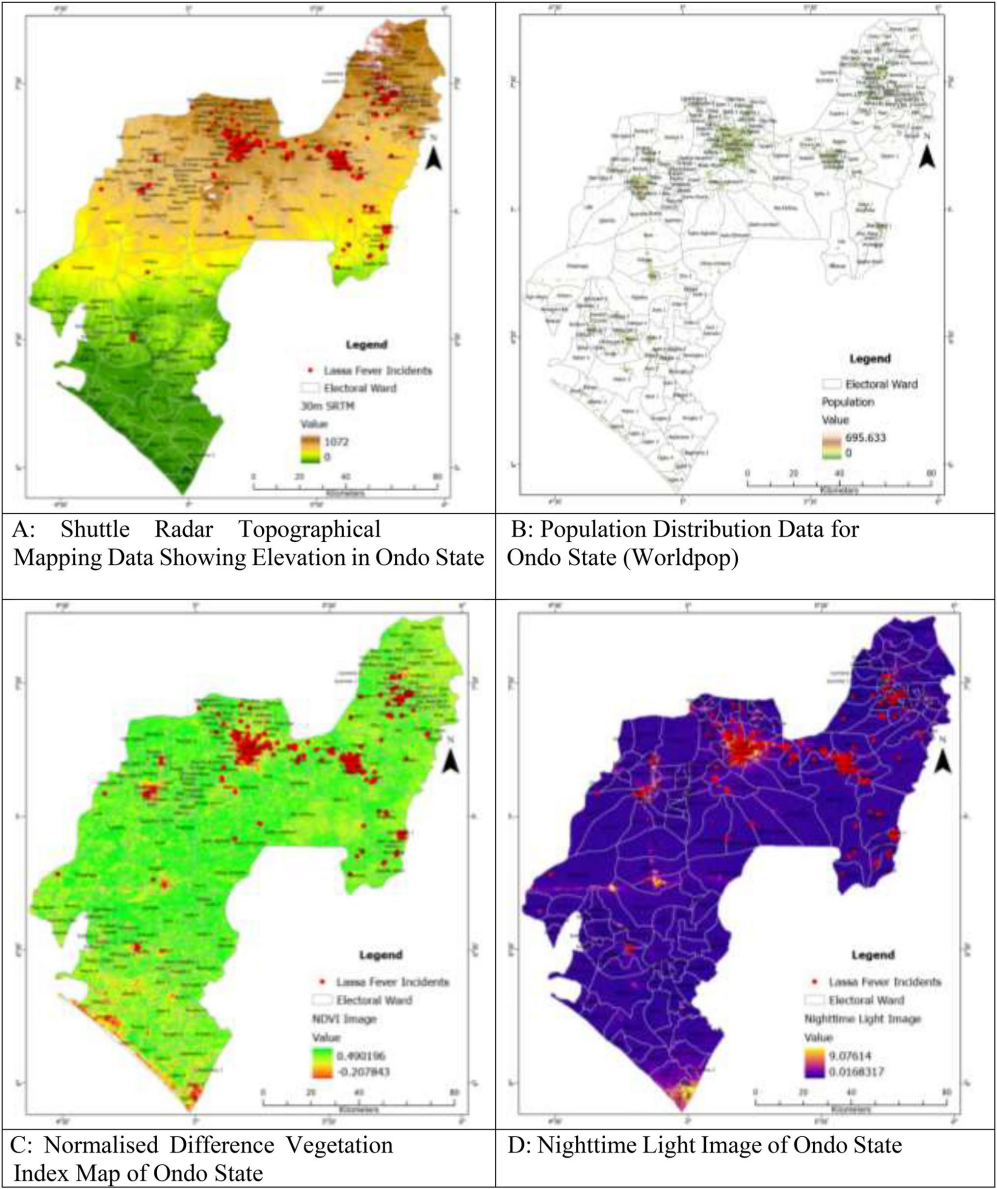

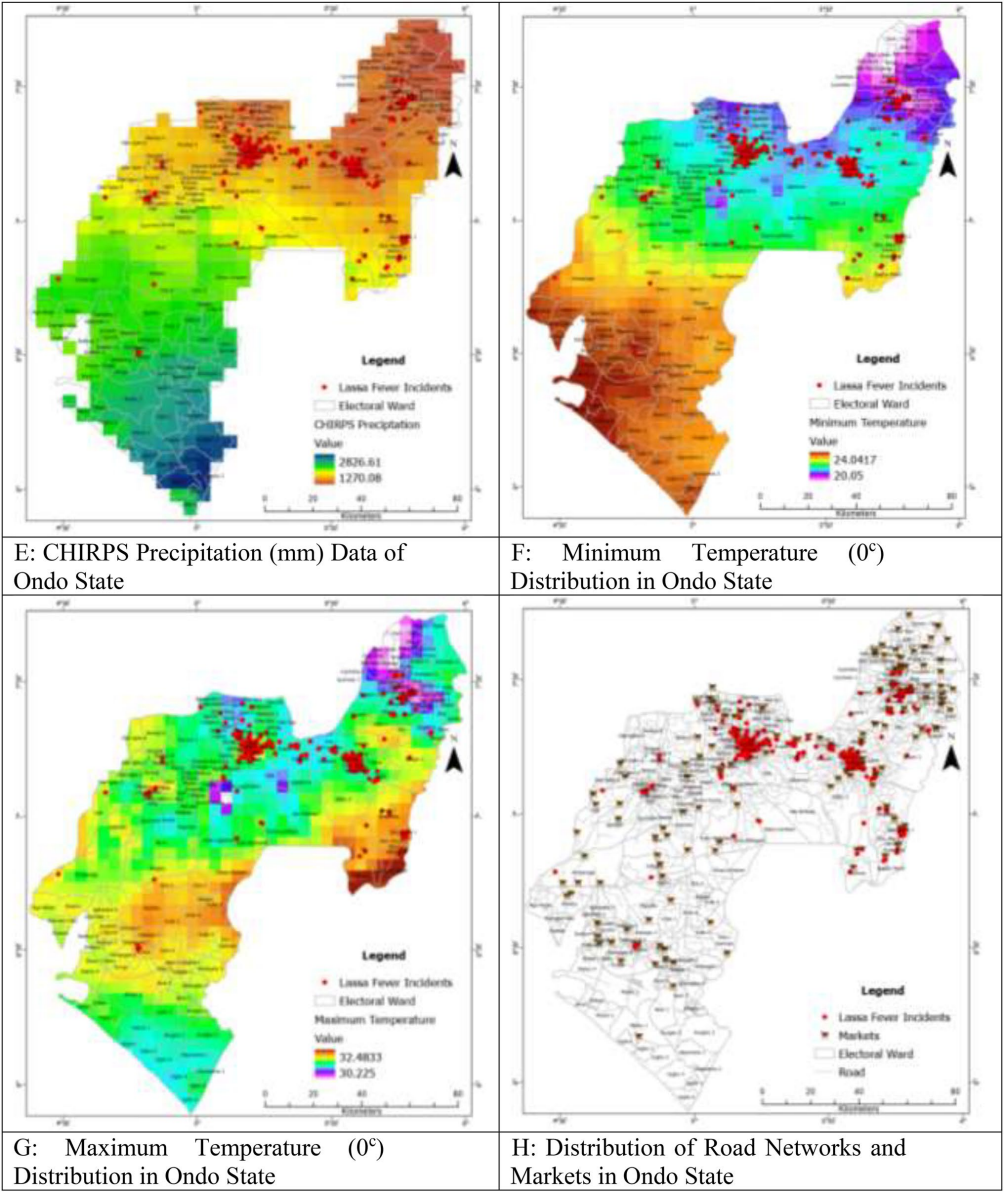

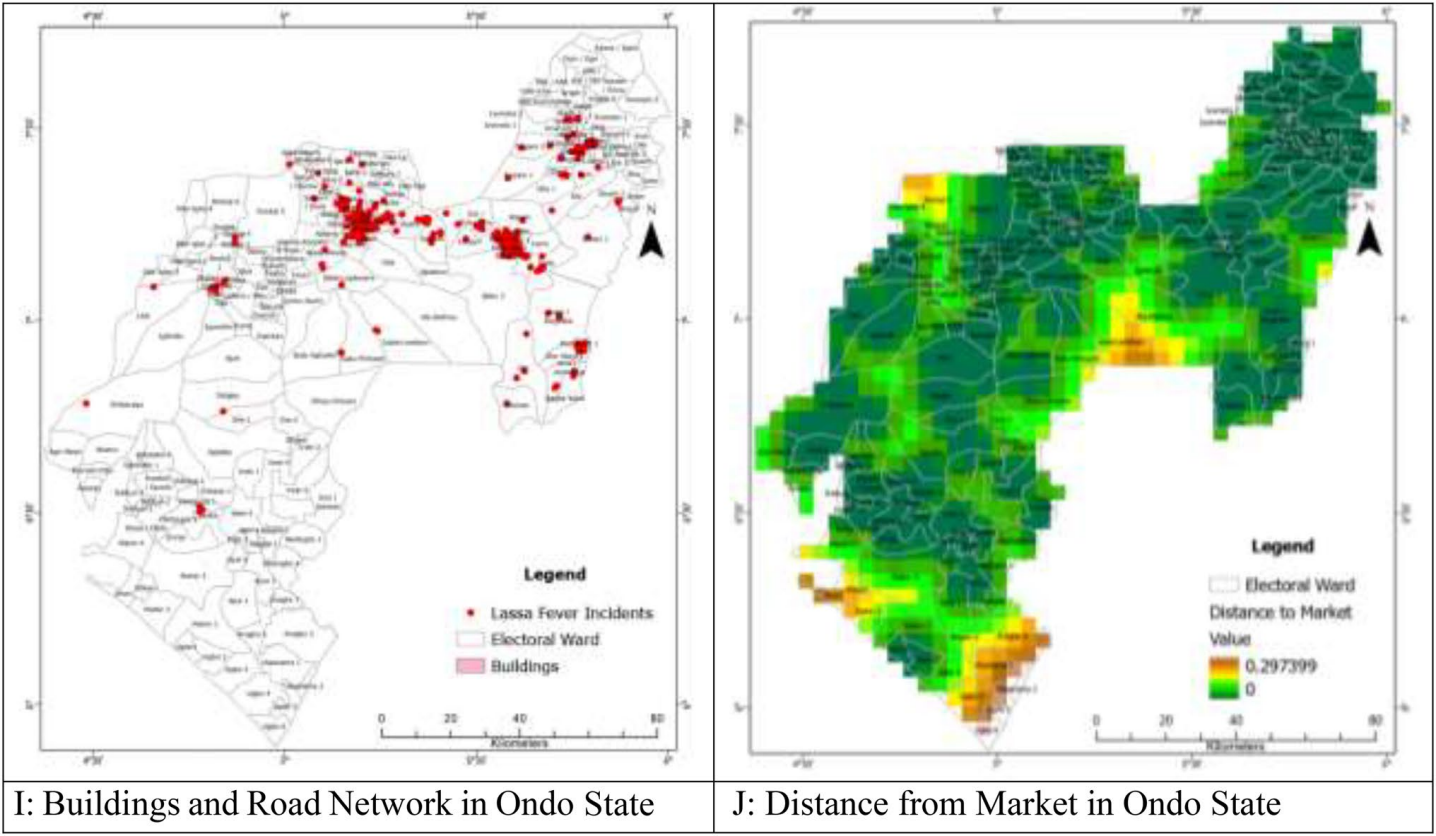
(**A**) Shuttle Radar Topographical Mapping Data Showing Elevation in Ondo State. (**B**) Population Distribution Data for Ondo State (Worldpop). (**C**) Normalised Difference Vegetation Index Map of Ondo State. (**D**) Nighttime Light Image of Ondo State. (**E**) CHIRPS Precipitation (mm) Data of Ondo State. (**F**) Minimum Temperature (0^c^) Distribution in Ondo State. (**G**) Maximum Temperature (0^c^) Distribution in Ondo State. (**H**) Distribution of Road Networks and Markets in Ondo State. (**I**) Buildings and Road Network in Ondo State. (**J**) Distance from Market in Ondo State.

In previous studies, researchers have explored the impact of elevation on the distribution of LF in the West African Sub-region^8^. For this study, the 30-m digital elevation data for Ondo State was downloaded from https://dwtkns.com/srtm30m/ in the form of four tiles. These tiles were then combined to create a seamless elevation dataset (Fig. 4A). Using ArcGIS Pro software, the average elevation for each ward in Ondo State was extracted through the *Zonal Statistic as Table function*. The extracted mean elevation values were subsequently linked to their respective wards. Additionally, road network data for Ondo State was obtained from https://www.openstreetmap.org/.

Due to the absence of recent census data in Nigeria, the 2021 gridded population data for Ondo State, acquired from the Worldpop database (worldpop.org), was used to analyse the impact of population dynamics on LF incidence. This raster population database provided estimated population figures for each ward, enabling the assessment of population contributions to LF (Fig. 4B).

*Mastomys* rodents inhabit not only homes but also bushy areas and farmland, which serve as suitable habitats for them. Consequently, more *Mastomys* are expected to be found in these bushy areas. The Normalised Difference Vegetation Index (NDVI) has been used to assess vegetation levels in various environments^30,33^. In this study, the NDVI image for Ondo State was obtained from https://land.copernicus.vgt.vito.be/, providing information about the greenness of the environment, scaled between − 1 and ± 1 (Fig. 4C). Additionally, the study explored the potential impact of Nighttime Light (NTL) on Mastomys' nocturnal activities. While NTL has been utilised in previous investigations^42,43^, its use in understanding the dynamics of LF is still being established. NTL imagery of Ondo State was obtained from https://eogdata.mines.edu/nighttime_light/annual/v20/2021/, based on Version 2.1 produced from monthly cloud-free average radiance^44^ (Fig. 4D).

The distribution of LF in endemic areas is influenced by climatic variables such as temperature and rainfall. However, integrating climatic data into LF modeling in Nigeria is challenging due to the scarcity and resolution of meteorological stations. To address this, rainfall data from the Climate Hazards Group InfraRed Precipitation with Station data (CHIRPS) was obtained. CHIRPS is a 35-year quasi-global rainfall dataset that combines satellite imagery and in-situ station data to create gridded rainfall time series. Average annual precipitation raster data (Fig. 4E) was downloaded from the CHIRPS website. Additionally, TerraClimate data, which combines high-spatial resolution climatological normals from the WorldClim dataset with time-varying data, was used for maximum (Fig. 4F) and minimum temperatures (Fig. 4G) analysis^8,45,46^.

Road network data for Ondo State was downloaded from the OpenStreetMap.org (Fig. 4H). *Mastomys* rodents also frequently inhabit residential houses, where they feed on various food residues and household wastes^47,48^. Housing data, including building footprints, were downloaded from https://minedbuildings.blob.core.windows.net/africa/nigeria.geojsonl.zip and clipped using the administrative boundary of Ondo State (Fig. 4I). The building footprints were generated from satellite imagery using machine learning and are licensed by Microsoft under the Open Data Commons Open Database License (ODbL).

*Mastomys* rodents are commonly found in unhygienic food markets due to poor environmental conditions and food residues^32,33^. Their faecal droppings and urine are often observed in food stores during early mornings. To analyse their spatial distribution, the coordinate locations of verifiable markets in Ondo State were obtained from https://grid3.org/ as a shape-file, and this was transformed to a distance surface to be able to assess the effect of distance from markets on LF incident (Fig. 4J).

### Data analysis

LF incidence analysis in this study specifically considered confirmed cases from hospitals and excluded suspected cases. Using ArcGIS Pro 3.0 software, laboratory confirmed LF cases were plotted to visualise the distribution of incidents from 2017 to 2021. Graphical and statistical inference methods were employed to examine LF incident distribution among Ondo State wards. The number of LF cases were aggregated based on the ward boundaries, providing data on the occurrences within each ward between 2017 and 2021.

The temporal change in confirmed LF incidents was analysed using a regression trend line in Microsoft Excel. The spatial pattern of LF incidents from 2017 to 2021 was explored using Average Nearest Neighbour (Rn) analysis. Rn values close to zero indicate clustering, 1 represents randomness, and > 1 represents regularity. Kernel Density Estimation (KDE) was used to identify LF clusters, which is a mathematical process to estimate the probability density of a variable. The KDE of LF incidents was implemented in ArcGIS Pro 3.0.

The study involved computing the number of LF incidents in each administrative ward in Ondo State by overlaying the ward boundary on the LF incidents map. This approach was also applied to extract data from multiple layers, such as elevation, population, precipitation, nighttime light image (NLT), vegetation (NDVI), temperature, market, distance from market, number of buildings, length of roads, and building area coverage. To address the issue of high collinearity among these variables, Principal Component Analysis (PCA) was utilised to reduce the dimensionality and generate uncorrelated variables. The PCA resulted in three factor scores, which were then employed in subsequent regression models including Ordinary Least Square (OLS), Geographical Weighted Regression (GWR), and Multiscale Geographical Weighted Regression (MGWR). GWR was calculated within the MGWR framework to identify the most suitable model for the data. The study acknowledges the potential for multicollinearity in local statistical models and effectively manages it through the implementation of PCA^49–51^.

The study employed the global Moran's I index to examine the spatial pattern of LF among wards in Ondo State. The Moran's I index is a widely used indicator of spatial autocorrelation that assesses the relationship between feature locations (wards) and attribute values (number of LF incidents). The contiguity edges corners option with row standardisation was utilised to investigate the influence of ward contiguity on LF incidents. The Moran's I statistic coefficient ranges from − 1 to + 1, where positive and significant coefficients indicate clustering of similar values, negative and significant coefficients indicate dispersion, and a zero coefficient suggests a random process. The statistical significance of the global Moran's I index was determined using the conditional permutation method with 999 permutations. The Moran's I statistic z-score and p-value provide the statistical significance of the calculated index^21,52–54^.

The study examined the relationship between LF incidents at the ward level and 11 predictor variables using Pearson product-moment correlation. To identify predictors of LF in Ondo State, a multiscale geographical weighted regression (MGWR) was employed, which allows flexibility in modeling spatial phenomena. The preferred model was selected based on highest R-Square and Adjusted R-Square values and lowest Akaike's Information Criteria (AIC). Initially, an Ordinary Least Square (OLS) regression (global model) was developed, assuming constant predictor variables across wards. Subsequently, an MGWR model was created using optimal bandwidths obtained through a golden section search routine. The variables were standardised for comparison, and MGWR maps were generated to visualise predictors of LF across wards^49^.

The independent sample t-test method was employed to compare variables between wards that reported LF incidents and those that did not. The aim was to determine if there were significant differences in ecological and environmental variables. Subsequently, a binary logistic regression analysis was conducted to identify which urban ecological and environmental variables predict LF incidents in all wards of Ondo State^19,25^.

### Ethical consideration

Approval was received from the Surveillance and Epidemiology Unit of Ondo State Ministry of Health and the University of Ibadan/University College Hospital Institutional Review Board (UI/UCH/22/0305). This study involved secondary data analysis and there was no contact with the patients. Access was granted to the secondary data after all forms of identifiers that could reveal the patient's identity were removed. Appropriate steps were taken to ensure confidentiality and privacy as stated in the Helsinki Declaration^55^.

### Supplementary Information

Supplementary Information 1.

Supplementary Information 2.

Supplementary Information 3.

## Data Availability

All the data for this study will be made available on request. Contact for request of data from this study: simeonc5@gmail.com; Brainbox_taiwo@yahoo.com.
