# Supplementary material for: Ecological correlates and predictors of Lassa fever incidence in Ondo State, Nigeria 2017–2021: an emerging urban trend
Source: Sci Rep. 2023 Nov 27;13:20855. doi: 10.1038/s41598-023-47820-3 (PMC10682180; doi:10.1038/s41598-023-47820-3)
Supplement: Supplementary file 1 — Supplementary Information 1. [file 41598_2023_47820_MOESM1_ESM.docx]

Supplementary Plate 1: Google Earth Image of Owo Town With confirmed LF Overlay

Supplementary Plate 2: Google Earth Image of Akure Town With confirmed LF Overlay

| 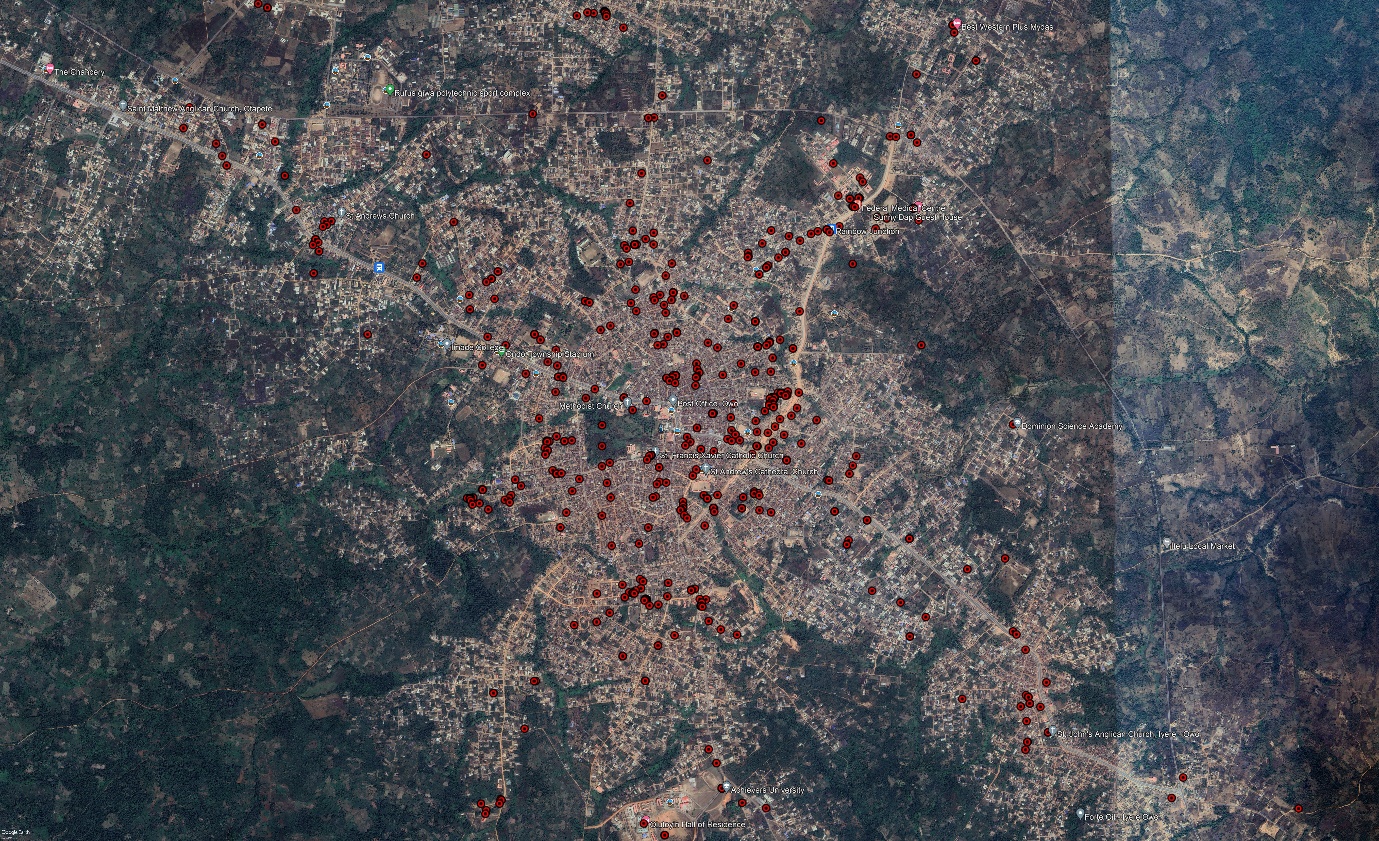 |
| --- |
| Plate 1: Google Earth Image of Owo Town With confirmed LF Overlay |
| 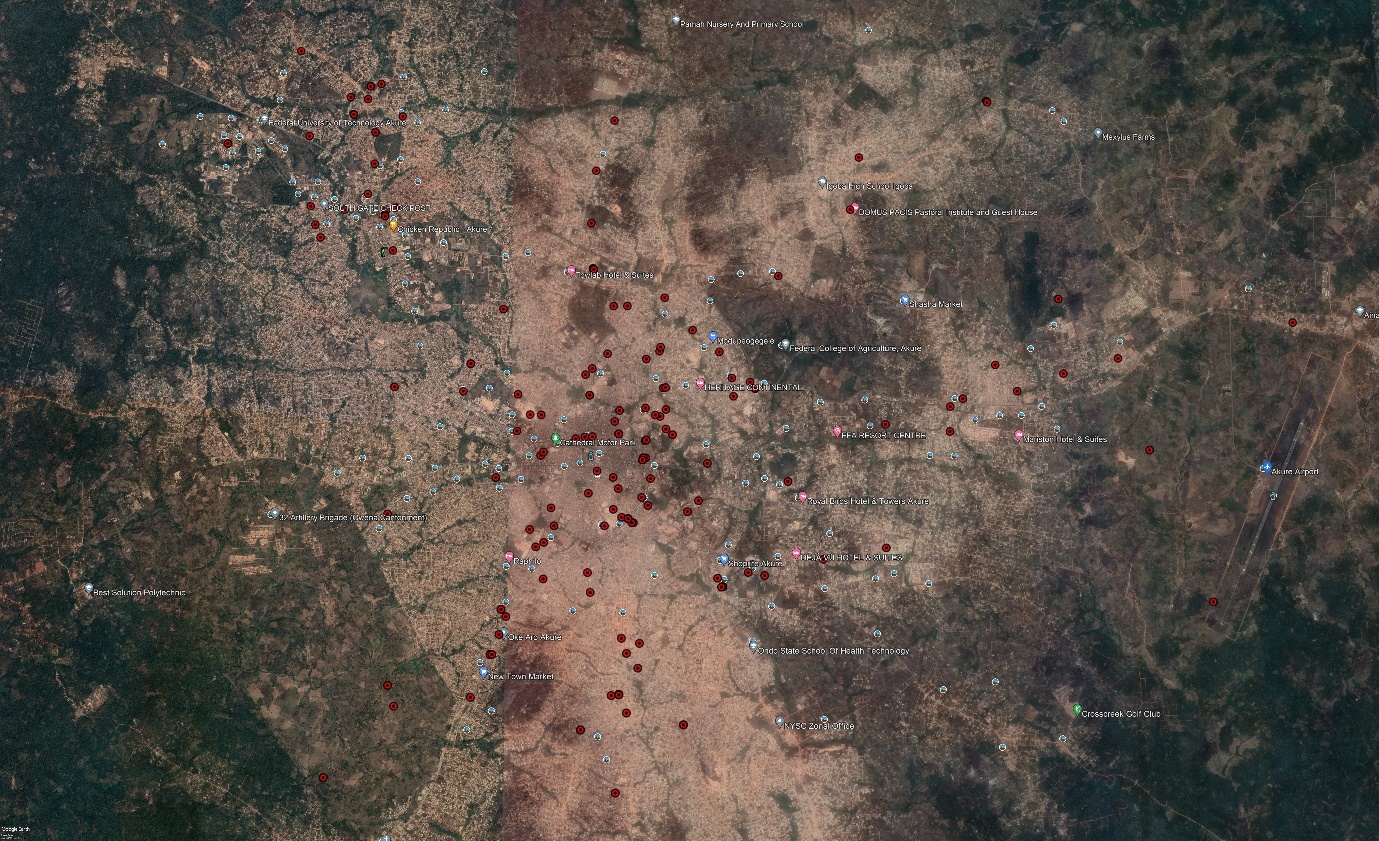 |
| Plate 2: Google Earth Image of Akure Town With confirmed LF Overlay |
