# Supplementary material for: Ecological correlates and predictors of Lassa fever incidence in Ondo State, Nigeria 2017–2021: an emerging urban trend
Source: Sci Rep. 2023 Nov 27;13:20855. doi: 10.1038/s41598-023-47820-3 (PMC10682180; doi:10.1038/s41598-023-47820-3)
Supplement: Supplementary file 2 — Supplementary Information 2. [file 41598_2023_47820_MOESM2_ESM.docx]

Supplementary Figure 1: Temporal Trend in Confirmed LF Incidents in Ondo State, 2017-2021

Supplementary Figure 2A: Wards with Significant Hotspot of LF in 2017

Supplementary Figure 2B: Wards with Significant Hotspot of LF in 2018

Supplementary Figure 2C: Wards with Significant Hotspot of LF in 2019

Supplementary Figure 2D: Wards with Significant Hotspot of LF in 2020

Supplementary Figure 2E: Wards with Significant Hotspot of LF in 2021

Supplementary Figure 2F: Wards with Significant Hotspot of LF between 2017 and 2021

Supplementary Figure 3A: MSGWR Coefficient of Human Factor

Supplementary Figure 3B: MSGWR Coefficient of Physical Factor

Supplementary Figure 3C: MSGWR Coefficient of Environmental Factor

Supplementary Figure 3D: MSGWR Coefficient of Regression Intercept

Supplementary Figure 3E: Wards with strongly positive association between LF incidence and Human and Environmental Factors

Supplementary Figure 1: Temporal Trend in Confirmed LF Incidents in Ondo State, 2017-2021

| 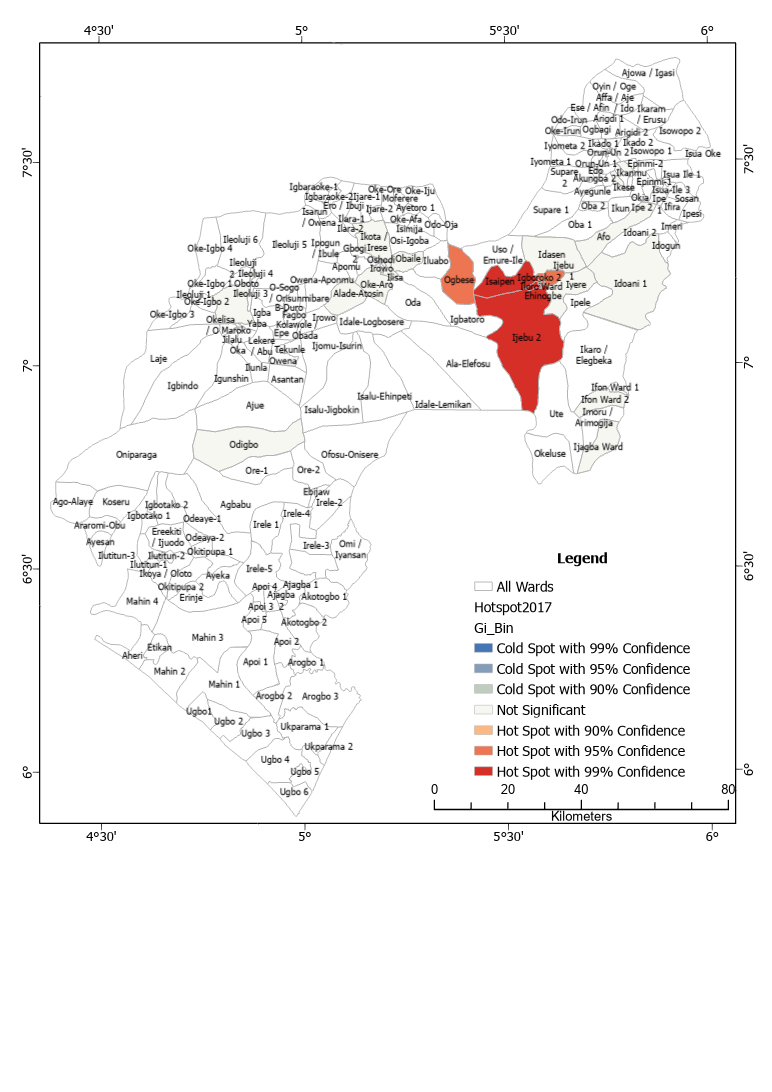 | 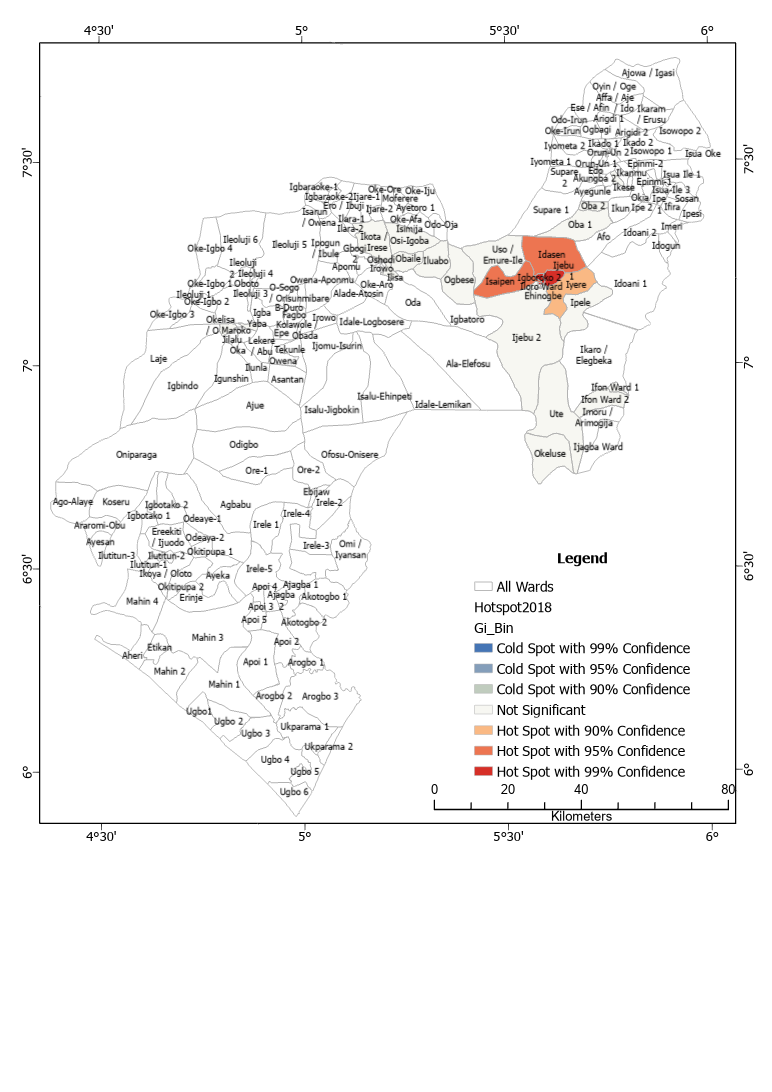 |
| --- | --- |
| Supplementary Figure 2A: Wards with Significant Hotspot of LF in 2017 | Supplementary Figure 2B: Wards with Significant Hotspot of LF in 2018 |
| 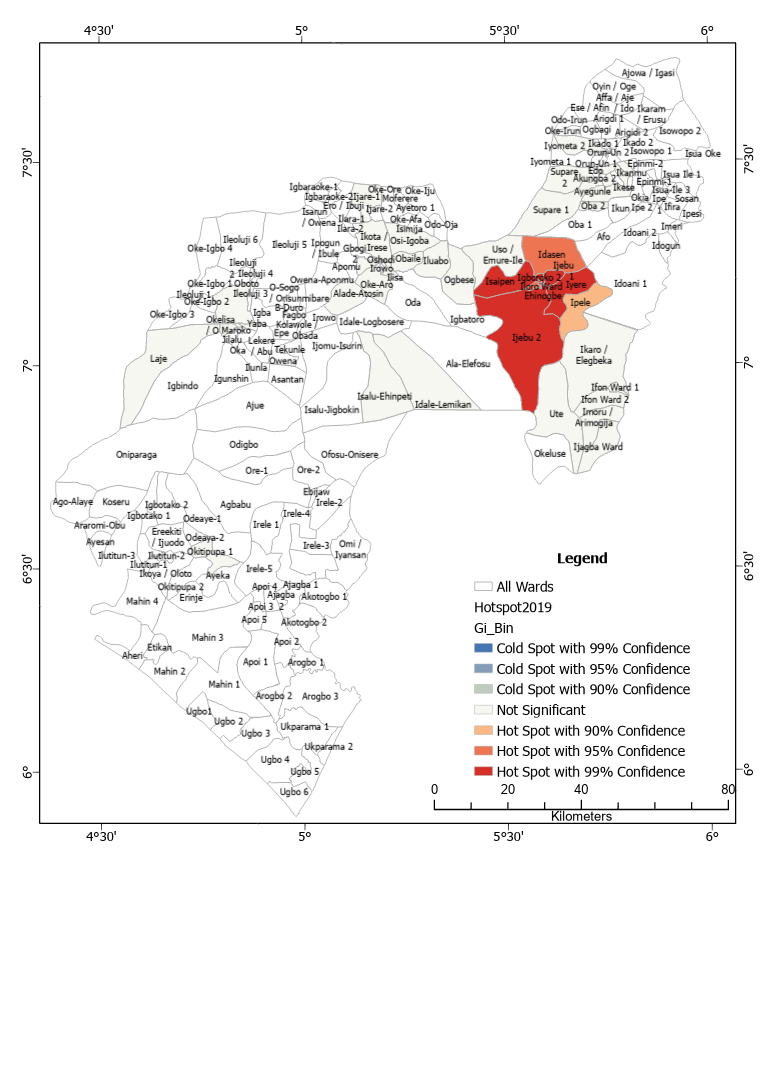 | 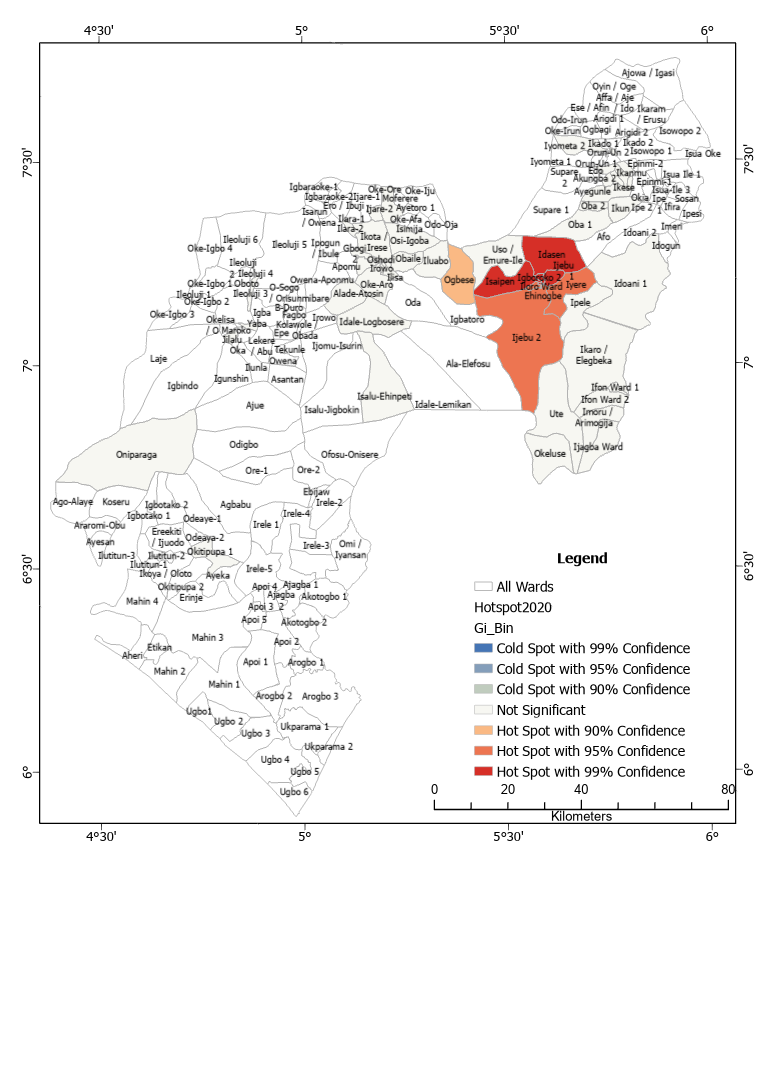 |
| Supplementary Figure 2C: Wards with Significant Hotspot of LF in 2019 | Supplementary Figure 2D: Wards with Significant Hotspot of LF in 2020 |
| 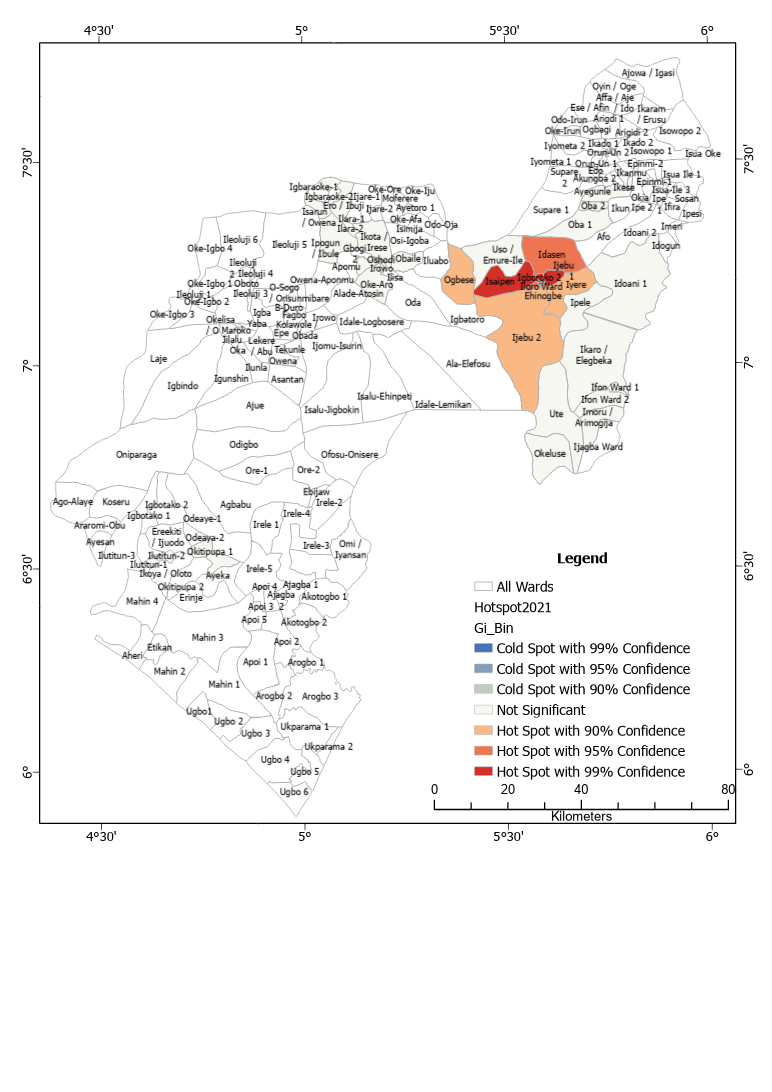 | 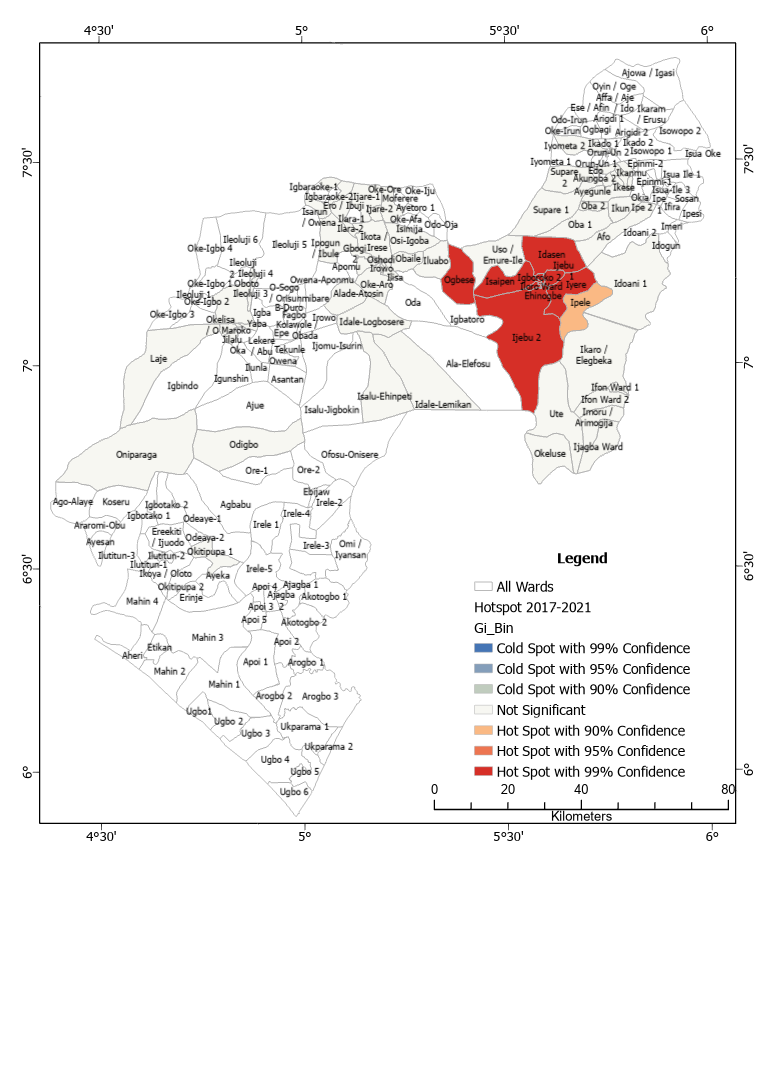 |
| Supplementary Figure 2E: Wards with Significant Hotspot of LF in 2021 | Supplementary Figure 2F: Wards with Significant Hotspot of LF between 2017 and 2021 |

| 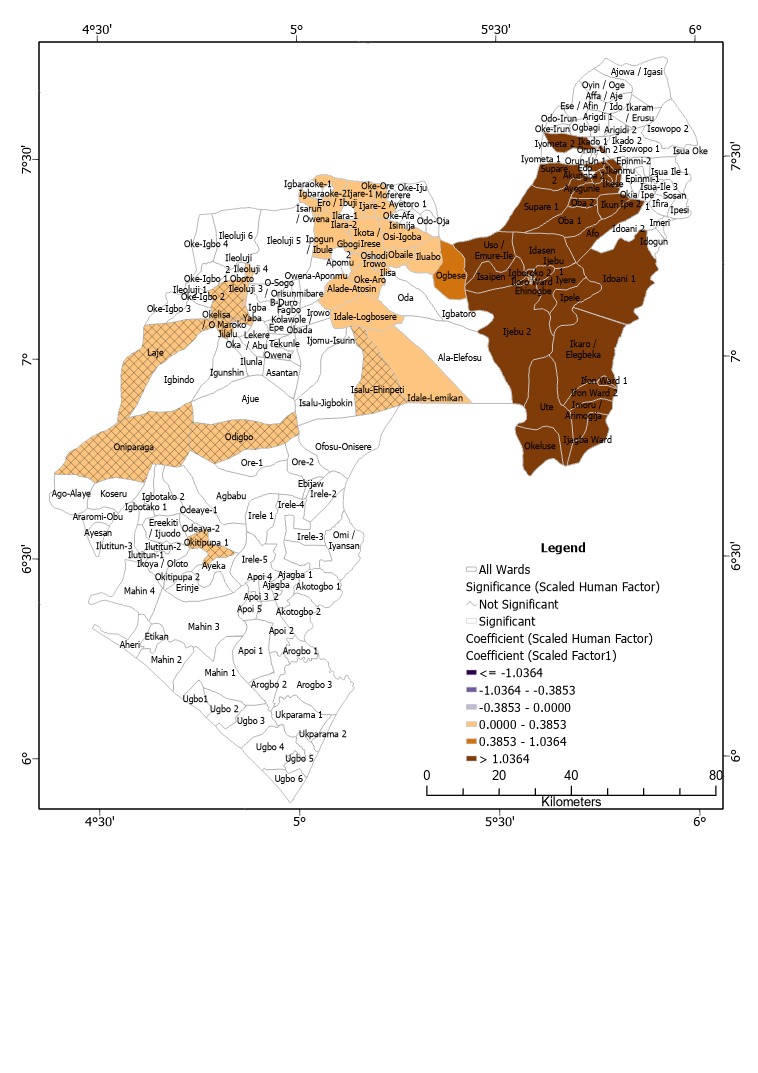 | 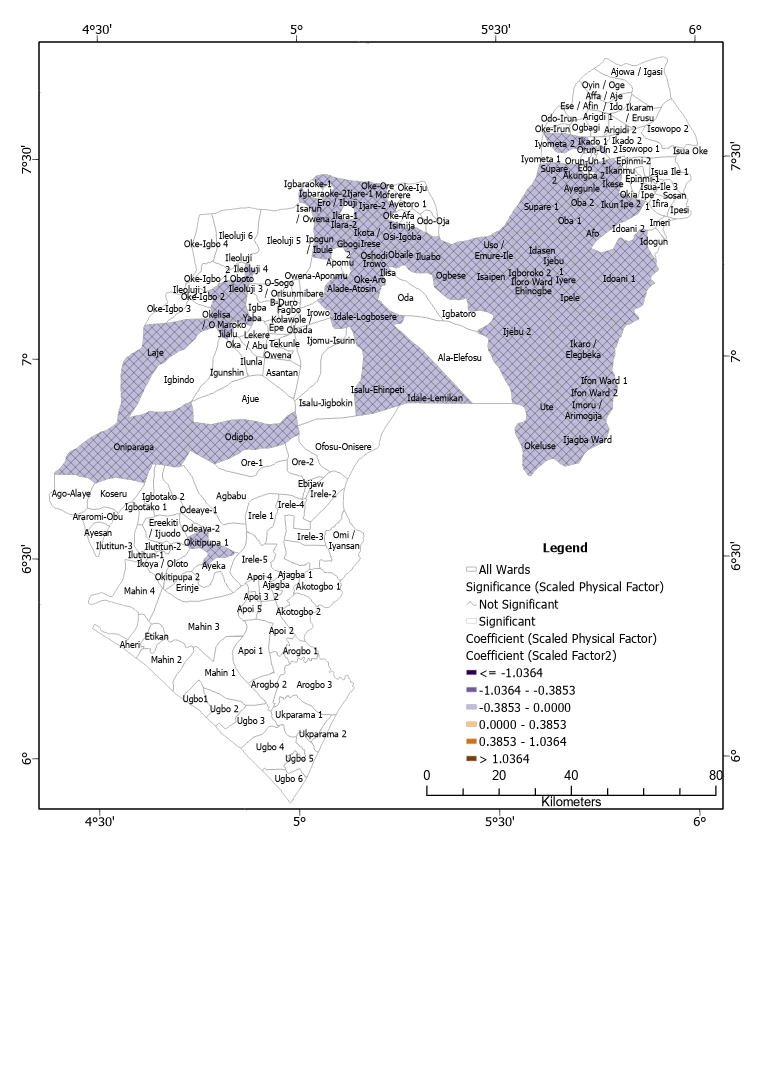 |
| --- | --- |
| Supplementary Figure 3A: MSGWR Coefficient of Human Factor | Supplementary Figure 3B: MSGWR Coefficient of Physical Factor |
| 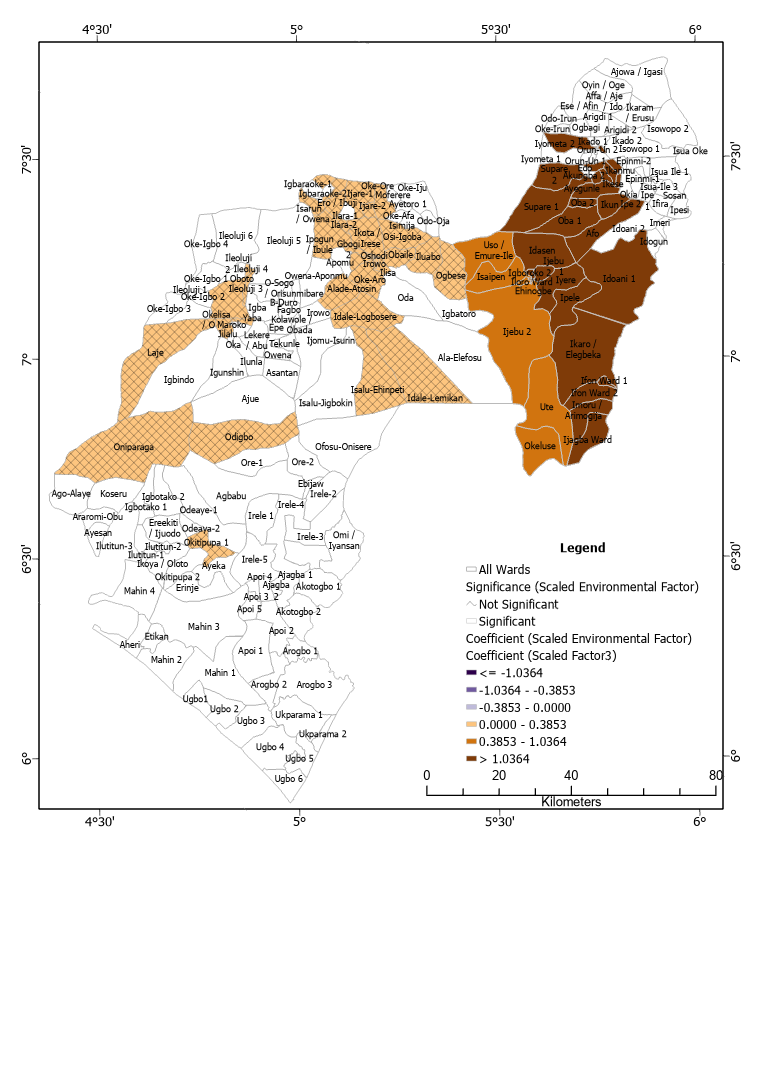 | 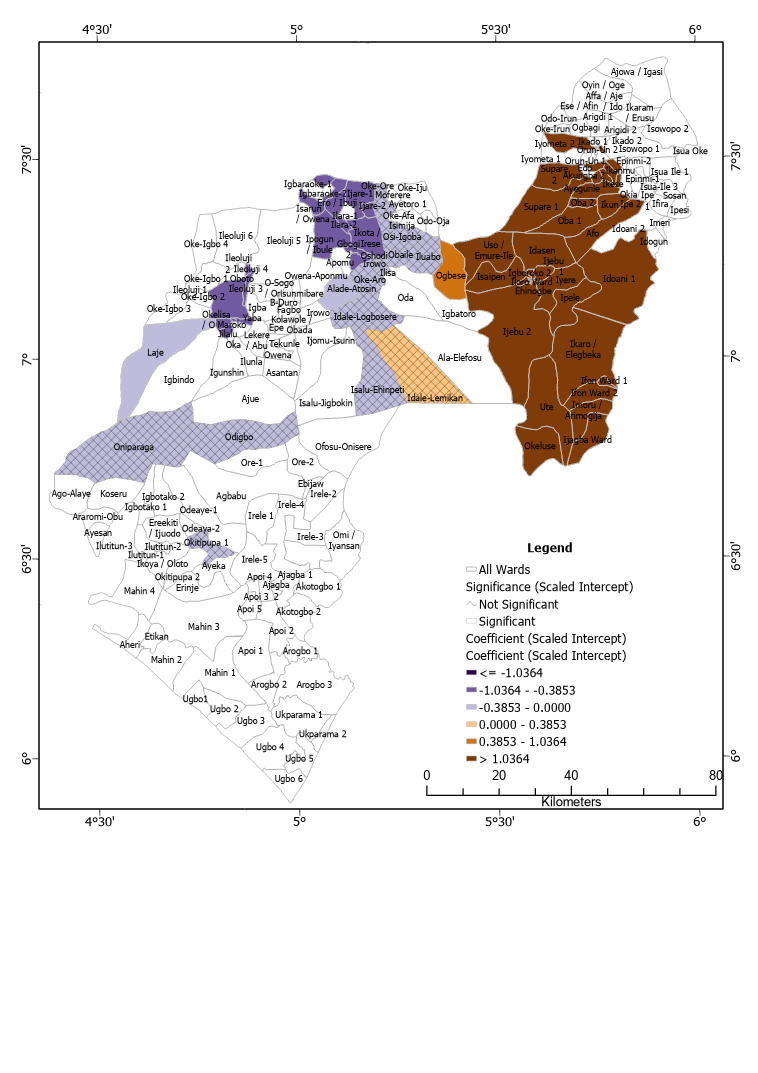 |
| Supplementary Figure 3C: MSGWR Coefficient of Environmental Factor | Supplementary Figure 3D: MSGWR Coefficient of Regression Intercept |
| 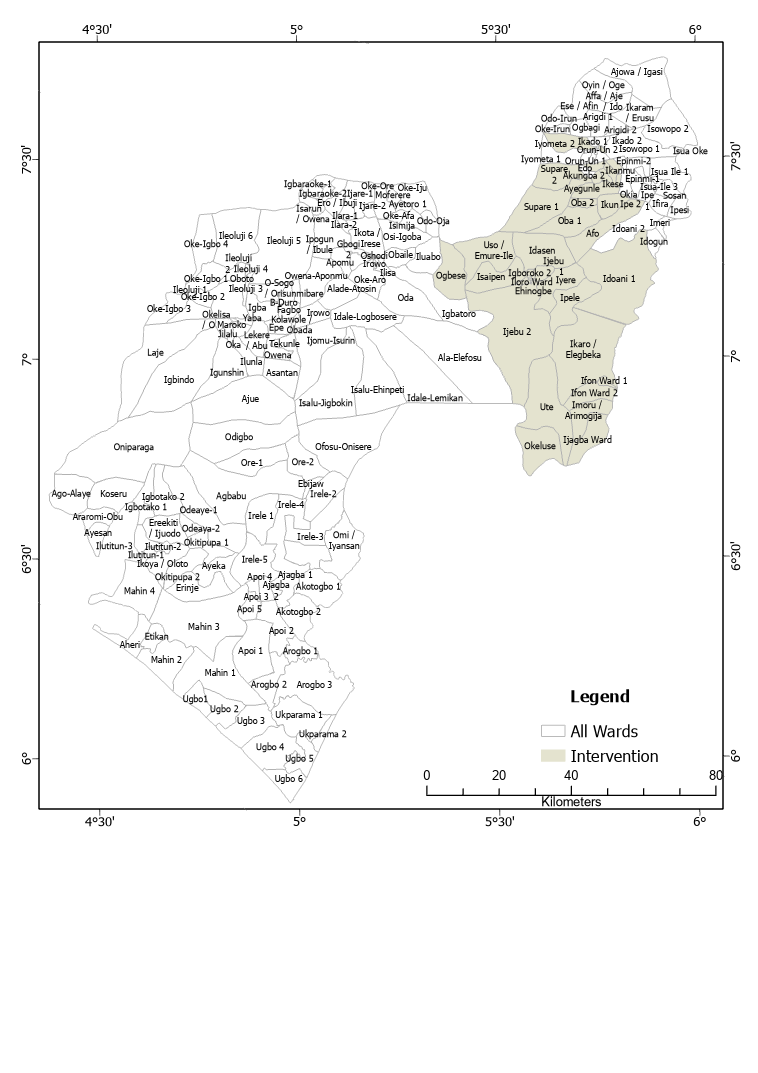 |  |
| Supplementary Figure 3E: Wards with strongly positive association between LF incidence and Human and Environmental Factors. |  |
