# Supplementary material for: Ecological correlates and predictors of Lassa fever incidence in Ondo State, Nigeria 2017–2021: an emerging urban trend
Source: Sci Rep. 2023 Nov 27;13:20855. doi: 10.1038/s41598-023-47820-3 (PMC10682180; doi:10.1038/s41598-023-47820-3)
Supplement: Supplementary file 3 — Supplementary Information 3. [file 41598_2023_47820_MOESM3_ESM.docx]

**Supplementary Table 1: Spatiotemporal Change in the Incidence of Lassa Fever in Ondo Between 2017 and 2021**

| Ward Name | LGA Name | LF2017 | LF2018 | LF2019 | LF2020 | LF2021 | Total |
| --- | --- | --- | --- | --- | --- | --- | --- |
| Igboroko 2 | Owo | 4 | 25 | 32 | 72 | 27 | 160 |
| Ijebu 2 | Owo | 5 | 18 | 29 | 42 | 20 | 114 |
| Ijebu 1 | Owo | 4 | 18 | 24 | 36 | 15 | 97 |
| Ehinogbe | Owo | 7 | 12 | 17 | 23 | 11 | 70 |
| Isaipen | Owo | 5 | 13 | 10 | 31 | 8 | 67 |
| Iyere | Owo | 2 | 6 | 8 | 10 | 6 | 32 |
| Ipele | Owo | 0 | 2 | 10 | 13 | 3 | 28 |
| Iloro Ward | Owo | 4 | 2 | 6 | 7 | 5 | 24 |
| Igboroko 1 | Owo | 5 | 1 | 8 | 3 | 5 | 22 |
| Idasen | Owo | 2 | 6 | 4 | 3 | 6 | 21 |
| Uso / Emure-Ile | Owo | 0 | 1 | 1 | 10 | 4 | 16 |
| Ifon Ward 2 | Ose | 1 | 9 | 11 | 8 | 4 | 33 |
| Ifon Ward 1 | Ose | 0 | 2 | 5 | 3 | 0 | 10 |
| Imoru / Arimogija | Ose | 0 | 0 | 2 | 3 | 4 | 9 |
| Ikaro / Elegbeka | Ose | 0 | 0 | 2 | 3 | 1 | 6 |
| Ute | Ose | 0 | 1 | 1 | 2 | 1 | 5 |
| Ijagba Ward | Ose | 2 | 0 | 2 | 1 | 0 | 5 |
| Idoani 1 | Ose | 2 | 0 | 0 | 2 | 1 | 5 |
| Okeluse | Ose | 0 | 1 | 0 | 1 | 1 | 3 |
| Afo | Ose | 1 | 0 | 0 | 0 | 0 | 1 |
| Yaba | Ondo West | 0 | 1 | 2 | 2 | 0 | 5 |
| Okelisa / O | Ondo West | 0 | 1 | 1 | 0 | 0 | 2 |
| Surulere | Ondo West | 1 | 0 | 0 | 0 | 0 | 1 |
| Laje | Ondo West | 0 | 0 | 1 | 0 | 0 | 1 |
| Jilalu | Ondo West | 0 | 0 | 0 | 1 | 0 | 1 |
| Okitipupa 1 | Okitipupa | 0 | 0 | 1 | 1 | 1 | 3 |
| Odigbo | Odigbo | 1 | 0 | 0 | 0 | 0 | 1 |
| Oniparaga | Odigbo | 0 | 0 | 0 | 1 | 0 | 1 |
| Ileoluji 1 | Ile Oluji/Okeigbo | 1 | 0 | 4 | 0 | 0 | 5 |
| Ileoluji 4 | Ile Oluji/Okeigbo | 1 | 0 | 0 | 0 | 0 | 1 |
| Ileoluji 3 | Ile Oluji/Okeigbo | 0 | 0 | 0 | 1 | 0 | 1 |
| Ikota / Irese | Ifedore | 3 | 7 | 20 | 30 | 8 | 68 |
| Ijare-1 | Ifedore | 0 | 0 | 1 | 0 | 0 | 1 |
| Ilara-2 | Ifedore | 0 | 0 | 0 | 1 | 0 | 1 |
| Ijare-2 | Ifedore | 0 | 0 | 0 | 1 | 0 | 1 |
| Isarun / Owena | Ifedore | 0 | 0 | 0 | 0 | 1 | 1 |
| Ero / Ibuji | Ifedore | 0 | 0 | 0 | 0 | 1 | 1 |
| Ipogun / Ibule | Ifedore | 0 | 0 | 0 | 0 | 1 | 1 |
| Alade-Atosin | Idanre | 1 | 0 | 1 | 2 | 0 | 4 |
| Idale-Lemikan | Idanre | 0 | 0 | 3 | 0 | 0 | 3 |
| Isalu-Ehinpeti | Idanre | 0 | 0 | 2 | 1 | 0 | 3 |
| Idale-Logbosere | Idanre | 0 | 0 | 0 | 1 | 0 | 1 |
| Oke-Aro | Akure South | 1 | 0 | 7 | 12 | 6 | 26 |
| Ilisa | Akure South | 1 | 2 | 2 | 10 | 0 | 15 |
| Odopetu | Akure South | 1 | 0 | 1 | 7 | 2 | 11 |
| Owode | Akure South | 0 | 2 | 0 | 1 | 1 | 4 |
| Gbogi 1 | Akure South | 0 | 0 | 2 | 1 | 1 | 4 |
| Ijomu | Akure South | 0 | 0 | 1 | 3 | 0 | 4 |
| Oshodi | Akure South | 0 | 2 | 0 | 0 | 1 | 3 |
| Irowo | Akure South | 0 | 0 | 0 | 3 | 0 | 3 |
| Gbogi 2 | Akure South | 0 | 0 | 0 | 1 | 0 | 1 |
| Ogbese | Akure North | 3 | 5 | 13 | 7 | 8 | 36 |
| Obaile | Akure North | 1 | 2 | 3 | 7 | 1 | 14 |
| Osi-Igoba | Akure North | 0 | 1 | 2 | 3 | 0 | 6 |
| Iluabo | Akure North | 0 | 1 | 2 | 1 | 0 | 4 |
| Oke-Ore | Akure North | 0 | 0 | 0 | 1 | 0 | 1 |
| Ikese | Akoko Southwest | 0 | 0 | 7 | 21 | 4 | 32 |
| Ayegunle | Akoko Southwest | 0 | 0 | 5 | 2 | 3 | 10 |
| Oka-Odo | Akoko Southwest | 1 | 4 | 0 | 2 | 0 | 7 |
| Agba | Akoko Southwest | 1 | 0 | 3 | 3 | 0 | 7 |
| Oba 2 | Akoko Southwest | 0 | 1 | 1 | 3 | 1 | 6 |
| Owalusi | Akoko Southwest | 0 | 0 | 3 | 1 | 0 | 4 |
| Akungba 1 | Akoko Southwest | 0 | 0 | 2 | 2 | 0 | 4 |
| Ibaka | Akoko Southwest | 0 | 0 | 0 | 3 | 1 | 4 |
| Oba 1 | Akoko Southwest | 0 | 1 | 0 | 1 | 1 | 3 |
| Supare 2 | Akoko Southwest | 0 | 0 | 2 | 0 | 0 | 2 |
| Supare 1 | Akoko Southwest | 0 | 0 | 2 | 0 | 0 | 2 |
| Ikun | Akoko Southwest | 0 | 0 | 0 | 2 | 0 | 2 |
| Ilepa 2 | Akoko Northeast | 0 | 0 | 0 | 1 | 3 | 4 |
| Iyometa 2 | Akoko Northeast | 0 | 0 | 2 | 1 | 0 | 3 |

**Supplementary Table 2: OLS, GWR, and MGWR Coefficients**

| Parameters | OLS | GWR | MGWR |
| --- | --- | --- | --- |
| R-Square | 0.124 | 0.6122 | 0.5779 |
| Adjusted R-Square | 0.110 | 0.4875 | 0.5054 |
| AICc | n/a | 171.0002 | 165.1737 |

**Supplementary Table 3: Model Diagnostics for GWR and MGWR**

| Statistic | GWR | MGWR |
| --- | --- | --- |
| R-Squared | 0.6122 | 0.5779 |
| Adjusted R-Squared | 0.4875 | 0.5054 |
| AICc | 171.0002 | 165.1737 |
| Sigma-Squared | 0.5102 | 0.4934 |
| Sigma-Squared MLE | 0.3878 | 0.4221 |
| Effective Degrees of Freedom | 53.204 | 59.8783 |

*The Optimal GWR Bandwidth of 34 neighbours was used (K nearest neighbours).*
